# A unique dual acyltransferase system shared in the polyketide chain initiation of kidamycinone and rubiflavinone biosynthesis

**DOI:** 10.3389/fmicb.2023.1274358

**Published:** 2023-10-27

**Authors:** Kyung Taek Heo, Byeongsan Lee, Gwi Ja Hwang, Beomcheol Park, Jun-Pil Jang, Bang Yeon Hwang, Jae-Hyuk Jang, Young-Soo Hong

**Affiliations:** ^1^Chemical Biology Research Center, Korea Research Institute of Bioscience and Biotechnology (KRIBB), Cheongju-si, Republic of Korea; ^2^College of Pharmacy, Chungbuk National University, Cheongju-si, Republic of Korea

**Keywords:** biosynthesis, natural products, polyketide synthase, dual acyltransferase, *Streptomyces*

## Abstract

The pluramycin family of natural products has diverse substituents at the C2 position, which are closely related to their biological activity. Therefore, it is important to understand the biosynthesis of C2 substituents. In this study, we describe the biosynthesis of C2 moieties in *Streptomyces* sp. W2061, which produces kidamycin and rubiflavinone C-1, containing anthrapyran aglycones. Sequence analysis of the loading module (Kid13) of the PKS responsible for the synthesis of these anthrapyran aglycones is useful for confirming the incorporation of atypical primer units into the corresponding products. Kid13 is a ketosynthase-like decarboxylase (KSQ)-type loading module with unusual dual acyltransferase (AT) domains (AT_1-1_ and AT_1-2_). The AT_1-2_ domain primarily loads ethylmalonyl-CoA and malonyl-CoA for rubiflavinone and kidamycinone and rubiflavinone, respectively; however, the AT_1-1_ domain contributed to the functioning of the AT_1-2_ domain to efficiently load ethylmalonyl-CoA for rubiflavinone. We found that the dual AT system was involved in the production of kidamycinone, an aglycone of kidamycin, and rubiflavinone C-1 by other shared biosynthetic genes in *Streptomyces* sp. W2061. This study broadens our understanding of the incorporation of atypical primer units into polyketide products.

## Introduction

Angucycline polyketides are a group of natural products synthesized by polyketide synthases (PKSs), which have diverse structures and a broad variety of biological activities, such as antibacterial, antiviral, antifungal, and anticancer activities (Kharel et al., 2012; Katz and Baltz, 2016). In bacteria, aromatic polyketides are usually biosynthesized by type II PKSs through a ketosynthase (KS)/chain length factor (CLF) heterodimer (KS-CLF) that iteratively elongates a poly-β-ketone chain linked to an acyl carrier protein (ACP) (Staunton and Weissman, 2001; Hertweck et al., 2007). A typical KS-CLF is primed with an acetate unit through the decarboxylation of malonyl-ACP. While only a few type II PKSs that use other starter units have been identified, the use of different starter units could provide additional structurally diverse products (Moore and Hertweck, 2002; Hertweck, 2009; Lesnik et al., 2015). For example, doxorubicin is primed by a propionyl unit, oxytetracycline by a malonamyl unit, frenolicin by a butyryl unit, hedamycin by a hexaonly unit, and R1128s by butyryl, valeryl, or 4-methylvaleryl units (Binnie et al., 1989; Bibb et al., 1994; Bao et al., 1999; Marti et al., 2000; Bililign et al., 2004). Although the mechanism of attachment of starter units has been controversial in several case studies, one can speculate that the activation and transfer of these non-acetate starter units are mediated by crotonyl-CoA carboxylase/reductase, CoA ligases, 3-ketoacyl-ACP synthase III (KASIII), and an atypical acyltransferase (AT) (Bililign et al., 2004; Hertweck et al., 2007; Wilson and Moore, 2012).

**Figure 1:**
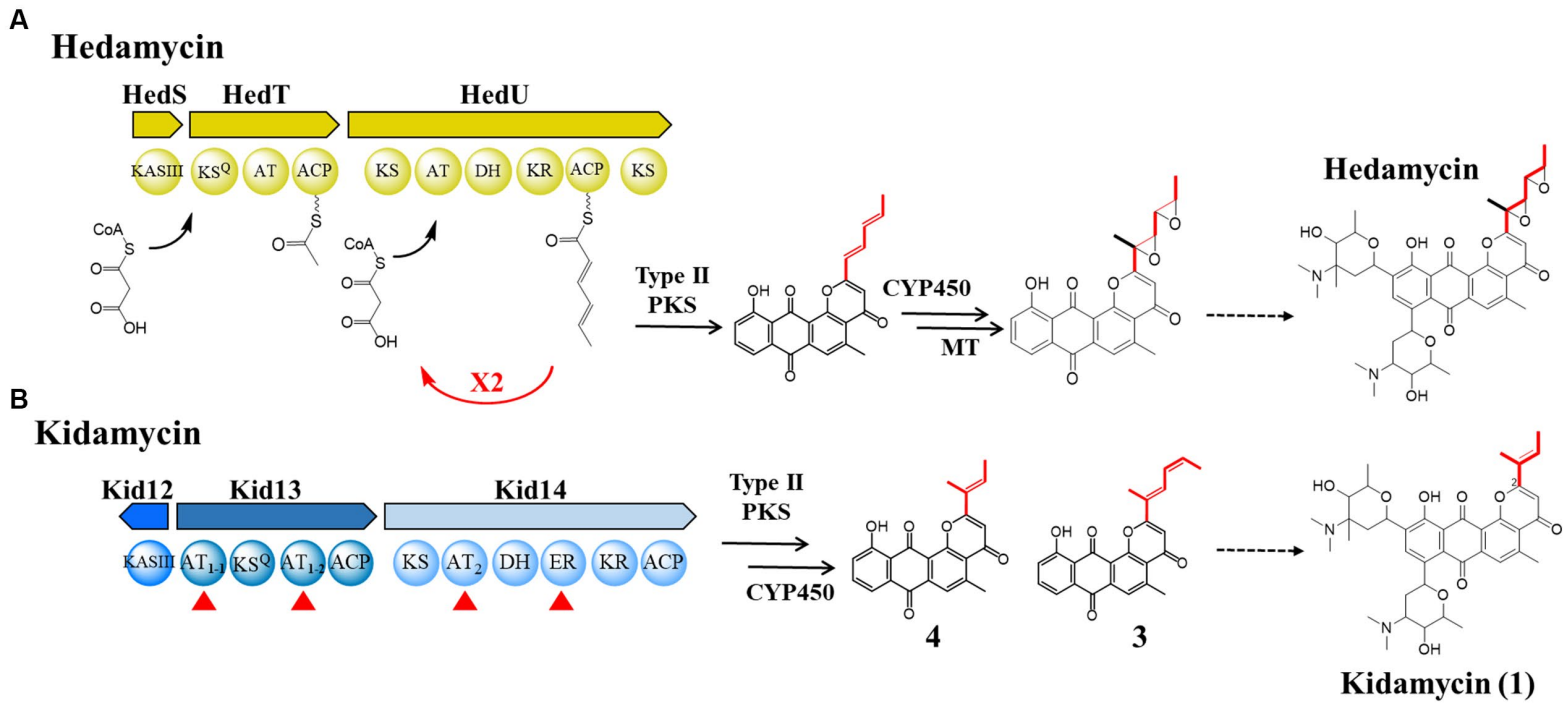
Comparison of the biosynthetic pathways of hedamycin and kidamycin. **(A)** Proposed mechanisms for chain initiation and elongation in the hedamycin PKS by Bililign et al. and Das et al. **(B)** Domain architecture of initiation (Kid13) and elongation (Kid14) modules in *kid* BGC for the kidamycin and rubiflavinone C-1. The sequences of homologous proteins in the two gene clusters were aligned; their identity/similarity is reported in Supplementary Table S4. AT, acyltransferase; KR, ketoreductase; DH, dehydratase; ER, enoyl reductase. The starter unit in each molecule is highlighted in red line.

The pluramycin family of angucycline antitumor antibiotics is a structurally complex DNA-reactive agent consisting of a planar anthrapyran substructure with variations in the side chain at C2 and substitution patterns of C-arylglycosidic deoxyaminosugars on the aglycone backbone (Schmitz et al., 1966; Umezawa et al., 1973; Cairns and Murray, 1998; Kharel et al., 2012; Lee et al., 2023). This unusual distal side chain implies the use of a non-acetate starter unit in anthrapyran aglycone biosynthesis. Among them, the *bis*-epoxide side chain of hedamycin is formed in the hybrid type I/type II PKS biosynthetic machinery (Bililign et al., 2004; Figure 1). Type I PKS acts iteratively to generate the hexadienyl starter unit that subsequently primes the partner type II PKS (Das and Khosla, 2009). In contrast, the hexadienyl side chain of fredericamycin, although it also synthesizes a similar six-carbon intermediate to hedamycin, is produced by the reduction of two-ketoacyl-ACP intermediates during two rounds of chain elongation via a three-ketoacyl ACP reductase (Das et al., 2010). Kidamycin (**1**), a member of the pluramycin family, has been derivatized in an attempt to increase its therapeutic index (Fei and Mcdonald, 2005, 2007; O’keefe et al., 2011; Mabit et al., 2017) and has a shorter four-carbon (2-methylbut-2-ene) side chain than hedamycin; however, its biosynthetic mechanism has not been reported.

Kidamycin (**1**) was first identified in *Streptomyces phaeoverticillatus* in 1971 (Kanda, 1971). It was subsequently isolated from several unidentified *Streptomyces* species. Compound **1** and its photoreactive derivative (**2**) showed promising anticancer activity against the triple-negative breast cancer cell line MDA-231 (Lee et al., 2023). Notably, our **1** producer, *Streptomyces* sp. W2061, also produces a de-epoxide hedamycin aglycone derivative, rubiflavinone C-1 (**3**), containing a hexadienyl side chain (Figure 1). It is estimated that the two types of compounds with different distal side chains will somehow be shared by type II PKSs involved in the backbone biosynthesis pathway, with an alternative starter unit for chain elongation. Previously, the biosynthetic gene cluster (BGC) of **1** (*kid*) was identified from the *Streptomyces* sp. W2061 (Heo et al., 2022). Our findings revealed that the *kid* BGC has a gene composition similar to that of the hedamycin BGC. Two type I PKS modules, similar to the hedamycin BGC, were also found; however, the domain architecture of each module did not completely match that of hedamycin. The first module (Kid13) contains two unusual acyltransferase (AT) domains (AT_1-1_ and AT_1-2_), and the second module (Kid14) carries an AT domain (AT_2_) with methylmalonyl-CoA-specific sequences and an additional enoylreductase (ER) domain (Figure 1). Based on heterologous expression and site-directed mutagenesis, the type I PKS system in *kid* BGC is responsible for the formation of both kidamycinone (**4**), an anthrapyran aglycone of **1**, and **3**. Furthermore, we propose that the AT_1-2_ domain mainly loads both malonyl-CoA and ethylmalonyl-CoA for **1** and **3**, but the AT_1-1_ domain acts in harmony to load ethylmalonyl-CoA efficiently for **3** by AT_1-2_ domain.

**Figure 2:**
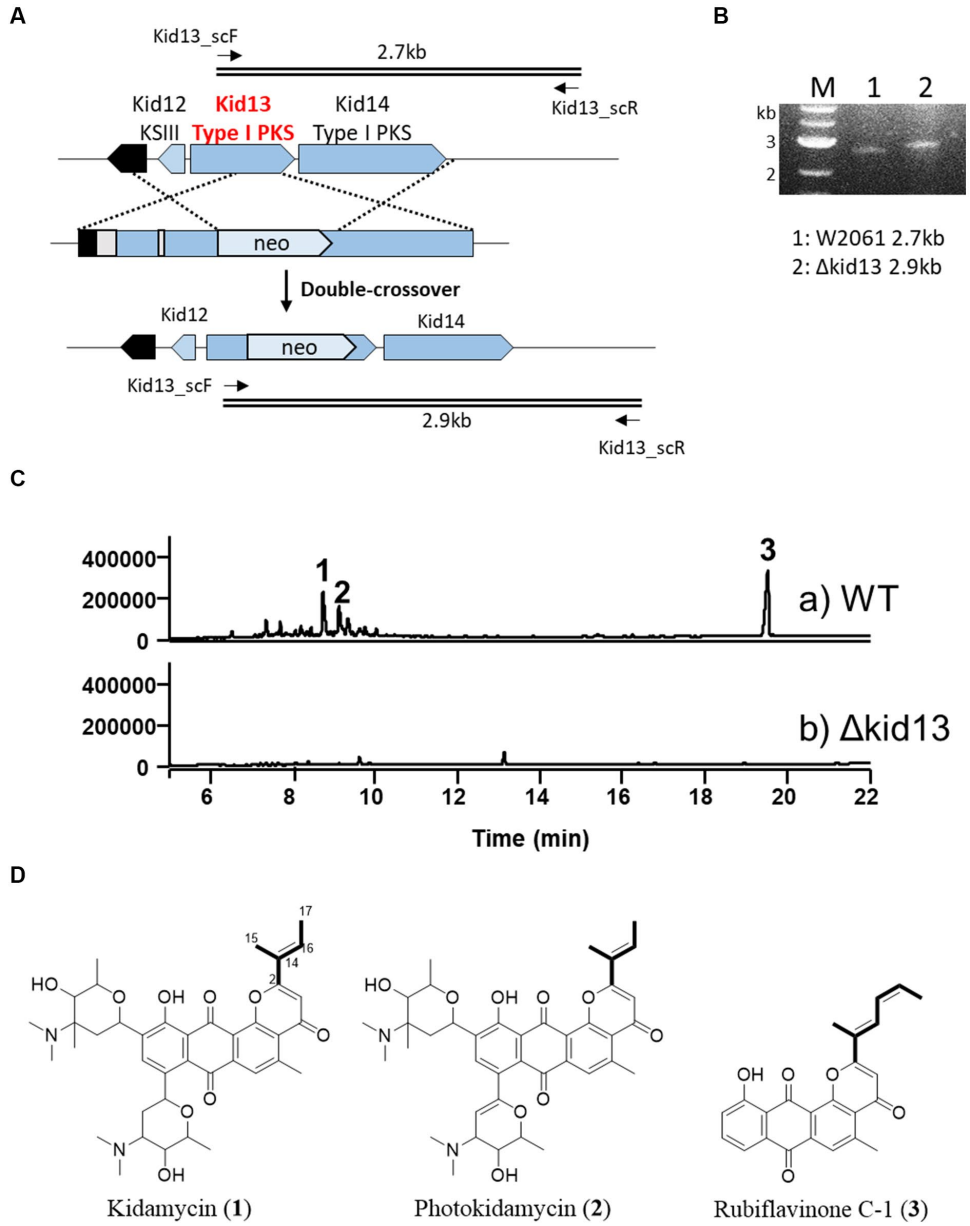
Gene disruption of type I PKS gene *kid13*. **(A)** Scheme representing the insertional inactivation of kid13 gene in Streptomyces sp. W2061. **(B)** Confirmation of insertional gene inactivation using PCR and the total genomic DNA of ∆Kid13 mutant as the template. The primers (kid13sc_F and kid13sc_R; Supplementary Table S1) used to amplify the desired DNA fragments are indicated by solid arrows. M, 1 kb ladder; 1, wild type; 2, ΔKid13 mutant. **(C)** Comparative HPLC profiles of extracts showing the wild type **(A)** and ΔKid13 **(B)**. Production levels are in the same order of magnitude. **(D)** Chemical structures of kidamycin (1), photokidamycin (2), and rubiflavinone C-1 (3) isolated from *Streptomyces* sp. W2061.

## Materials and methods

### General experimental procedures

Restriction enzymes (NEB, United States; Takara, Japan), KOD-plus-DNA polymerase (Toyobo, Japan), PrimeSTAR^®^ GXL DNA Polymerase (Takara, Japan), and a DNA ligation kit (Takara) were used according to the manufacturer’s instructions. The T-Blunt vector (BioFact, Daejeon, Korea) was used to clone the polymerase chain reaction (PCR) products. Antibiotics were added to the medium at the following concentrations: 50 μg/L apramycin, 50 μg/L kanamycin, and 25 μg/L chloramphenicol. Gene inactivation experiments were performed using the vector pKC1139, and the kanamycin resistance gene from pFD-NEO-S (Denis and Brzezinski, 1991) was used as the selection marker. *Escherichia coli* DH5α was used for plasmid cloning and amplification, and the ET12567/pUZ8002 strain was used to introduce the plasmid into the *Streptomyces* sp. W2061 strain via conjugation, and the ET12567/pUB307 strain was used to introduce the plasmid into the *Streptomyces albus* J1074 strain via tri-parental intergeneric conjugation according to the instructions given by Zhang et al. (2019).

### Culture conditions and extraction

*Streptomyces* sp. W2061 and mutant strains were grown in an ISP4 plate (10 g/L soluble starch, 1 g/L K_2_HPO_4_, 1 g/L MgSO_4_·7H_2_O, 1 g/L NaCl, 2 g/L (NH_4_)_2_SO_4_, 2 g/L CaCO_3_, 0.001 g/L FeSO_4_·7H_2_O, 0.001 g/L MnCl_2_·4H_2_O, 0.001 g/L ZnSO_4_·7H_2_O, and 15 g/L agar, pH 7.0–7.4) at 28°C for 4 days. Thereafter, they were inoculated into seed culture M2 medium (2 g/L yeast extract, 5 g/L glucose, 25 mL/L glycerol, 4 g/L soytone, 0.03 g/L CaCO_3_, and pH 7.2) and incubated for 2 days at 28°C, following which, 15 mL of seed culture was transferred to a 1-L flask containing 300 mL of M2X medium (M2 medium +5 g/L MgCO_3_) (Lee et al., 2023) and incubated for 5–7 days. For heterologous expression, the recombinant *S. albus* J1074 strain was grown in an ISP4 plate at 28°C for 4 days and subsequently inoculated into seed culture M2 medium. After 2 days at 28°C, 15 mL of the seed culture was added to a 1-L flask containing 300 mL of M2 medium with kanamycin and incubated at 28°C for 5 and 7 days. The culture broth was extracted using an equal volume of ethyl acetate. Thereafter, the ethyl acetate was dried and resuspended in methanol for high-performance liquid chromatography (HPLC) and liquid chromatography-mass spectrometry (LC-MS) analysis.

### Bioinformatics analysis

The genomic DNA of *Streptomyces* sp. W2061 was sequenced, and bioinformatics analysis data of the kidamycin biosynthetic gene cluster (*kid*) were registered in the GenBank database under the accession number ON993775. The amino acid sequences of AT_1-1_, AT_1-2_, AT_2_, and other ATs involved in the biosynthesis of natural products were aligned using Clustal W. Phylogenetic tree analysis using the minimum evolution method was based on the results of sequence alignment, and evolutionary distances were computed using the Poisson correction method in MEGA 7 software (Kumar et al., 2016).

### Cloning of the kidamycin minimal PKS region

To clone the minimal PKS region of kidamycin, we constructed a cosmid library of *Streptomyces* sp. W2061 using the Supercos1 vector kit and Gigapack III gold packaging extract (Agilent Technologies, CA, United States; #251301, #200201) and screened via PCR with appropriate primers (kid4-H1-F/kid4-H1-R and kidR1sc_F/kidR1sc_R) (Supplementary Table S1). A positive clone containing the kidamycin-minimal PKS region was obtained. The cosmid vectors were digested with HpaI for linearization and cloned into pCAP03-acc(3)IV (Tang et al., 2015) via transformation-associated recombination (TAR) in *Saccharomyces cerevisiae* BY4727 (Yamanaka et al., 2014). For TAR cloning, BY4727 was inoculated in 5 mL YPD (glucose 20 g/L, yeast extract 10 g/L, and peptone 20 g/L) from a plate colony and cultured at 30°C for 2 days, harvested, and washed with 1 mL LiAc/TE (0.1 M LiAc, 10 mM Tris-HCl pH 7.5, 1 mM EDTA). The harvested cells were resuspended in 0.5 mL of LiAc/TE, and 100 μL aliquoted cells were used for the TAR experiment. Specific capture arms were synthesized (Supplementary Table S1) and cloned into the pCAP03-acc(3)IV vector (digested with NdeI and XhoI) via Gibson assembly. Linearized cosmid and linearized specific capture vector with PacI were added to yeast cells with 50 μg salmon sperm DNA (Sigma-Aldrich, United States) and 0.6 mL PEG/LiAc (40% PEG 3350, 0.1 M LiAc, 10 mM Tris-HCl pH 7.5, 1 mM EDTA). The mixture was incubated at 30°C for 30 min, and 70 μL of DMSO was added to enhance the transformation efficiency. Heat shock was demonstrated at 42°C for 15 min and chilled on ice for 5 min. Finally, cells were resuspended in 200 μL of yeast synthetic tryptophan drop-out medium (TD) and spread on the TD plate, which contained 5-fluoroorotic acid 0.001% (w/v) (Zymo Research, CA, United States). Positive clones were selected by colony PCR using two primer pairs (kid4-H1-F/kid4-H1-R and kidR1sc_F/kidR1sc_R). Plasmids (pCAP03-kidm2) were isolated from the positive clone and transferred to *E. coli* DH5α. The constructs were amplified in *E. coli* and confirmed via restriction enzyme digestion (Supplementary Figure S1).

### *In vitro* CRISPR/Cas9-mediated gene editing

The reaction mixture (30 μL) containing 10 μg target plasmid (pCAP03-kidm2), 20 nM Cas9 protein (New England Biolabs, MA, United States), 3 μL NEB buffer 3.1, and 10 nM synthetic sgRNAs (Bioneer, Daejeon, Korea) were incubated overnight at 37°C. The digested plasmid was recovered from the agarose gel. Briefly, the gel fragment of interest was cut into 1–2 mm pieces and placed in an Eppendorf tube with an equal volume of phenol. The tube was placed at −80°C for 5 min and at room temperature (20°C) for 20 min. The mixture was centrifuged at 12,000 rpm for 15 min at room temperature. The supernatant was recovered and precipitated DNA by adding 1/10 volume of 5 M NaCl and 2.5 volume of cold 100% ethanol. The tube was placed at −80°C for 15 min and centrifuged at 12,000 rpm for 5 min. The pellet was washed with 70% ethanol, dried, and resuspended in water. To delete the AT_1-1_ domain, two sgRNAs (AT1-1D_sg1 and AT1-1D_sg2, Supplementary Table S1) were designed and synthesized (Bioneer, Daejeon, Korea) for double-strand breaking of both the 5′ and 3′ ends of the AT_1-1_ region. The plasmid with the target region removed was religated and transformed into *E. coli*. Positive clones were selected via colony PCR using appropriate primer pairs (AT1D_scF and AT1D_scR) and sequencing (Supplementary Figure S3). For site-directed mutagenesis of the AT_1-1_ and AT_1-2_ domains, four sgRNAs (AT1-1_sg1 and AT1-1_sg2 for AT_1-1_ and AT1-2_sg1 and AT1-2_sg2 for AT_1-2_, Supplementary Table S1) were designed, synthesized, and used for digestion with the Cas9 enzyme as mentioned above. Oligomers were designed to inactivate the AT domain by replacing the active serine site with alanine (AT1-1_A for AT_1-1_ and AT1-2_A for AT_1-2_). Oligomers were annealed with 1 μL of 10 × PCR buffer, incubated at 95°C for 10 min, and cooled down to 30°C for 20 min. CRISPR-Cas9 digested plasmid and annealed oligomers were mixed with Gibson Assembly Master mix (New England Biolabs), incubated at 50°C for 1 h, and transformed into *E. coli* according to the manufacturer’s protocol. Positive constructs were screened via digestion of PCR products amplified with appropriate primer pairs (AT1D_scF and AT1D_scR for AT_1-1_ inactivation and AT1-2_scF and AT1-2_scR for AT_1-2_ inactivation) (Supplementary Figure S3).

### Construction of gene disruption vectors and mutant strains

Gene knockout recombinants were generated using homologous recombination. The target region was replaced with a kanamycin resistance gene. The kanamycin resistance cassette was digested with KpnI and PstI from plasmid pFD-NEO-S (Supplementary Table S2). Two homologous regions were amplified using the appropriate primers (Supplementary Table S1). To inactivate *kid13*, a 1.4 kb EcoRI/KpnI fragment generated using kid13-H1-F and kid13-H1-R (Supplementary Table S1) and a 1.2 kb PstI/HindIII fragment generated using kid13-H2-F and Kid13-H2-R (Supplementary Table S1) were digested, ligated, and cloned in the EcoRI and HindIII sites of pKC1139 to yield pKC1139-Kid13H12neo. To generate a mutant strain, three fragments were ligated with plasmid pKC1139 and introduced into *Streptomyces* sp. W2061. The W2061 strain was conjugated with *E. coli* ET12567/pUZ8002. Exconjugants were selected based on antibiotic resistance (kanamycin and apramycin) and PCR genotyping. Double-crossover mutants were screened based on kanamycin resistance and PCR genotyping (kid13_scF/R for ∆Kid13 mutant) (Supplementary Table S1).

For deletion of the AT_1-1_ domain in the *Streptomyces* sp. W2061 strain, the pCRISPomyces-2 system was used (Cobb et al., 2014). The sequence of the two sgRNAs was the same as that used to generate the Kidm2-AT1D construct. The recombination template was obtained from kidm2-AT1D by PstI digestion and cloned into the T-Blunt vector PstI site (BioFact, Daejeon, Korea) to generate the AT1DH_TA construct. AT1DH_TA was digested with SpeI and XbaI and cloned into the pCRISPomyces-2 vector at the XbaI site. The final vector (pCRI-AT1D) was conjugated from *E. coli* ET12567/pUZ8002 to the *Streptomyces* sp. W2061 strain. Following conjugation, individual exconjugants were incubated on an R2YE liquid medium supplemented with 50 mg/mL apramycin at 28°C for 3 days. Clearance of the temperature-sensitive plasmid was accomplished with high-temperature cultivation (42°C) for 2–5 days, followed by replica plating on selective and non-selective plates to confirm loss of apramycin resistance. To determine the in-frame deletion, each selected apramycin-sensitive colony was subjected to PCR using appropriate primer pairs (AT1D_scF and AT1D_scR) (Supplementary Figure S4).

### LC-MS analysis

The samples were dissolved in methanol and analyzed using a Thermo U3000-LTQ XL ion trap mass spectrometer (Thermo Scientific, Waltham, MA, United States) equipped with an electrospray ionization (ESI) mass source. Chromatographic separation of the compounds was achieved using a Waters HSS T3 C18 column (2.1 × 150 mm, 2.5 μm) at a flow rate of 0.3 mL/min. The mobile phases A and B contained 0.1% formic acid, water, and acetonitrile, respectively. Gradient elution was performed as follows: 5–100% B for 0–15 min with a linear gradient, followed by 5 min of 100% B. The MS/MS system was operated in ESI mode. Mass spectra were acquired in the m/z range of 100–2,000 by applying three microscans and a maximum ion injection time of 100 ms. Data-dependent mass spectrometry experiments were controlled using the menu-driven software provided in the Xcalibur system (version 4.0; Thermo Scientific).

### Compound isolation

To isolate compounds **3**–**5**, the crude extract (2.0 g) of the Kidm2 mutant was fractionated using a CombiFlash RF (Teledyne ISCO, United States) medium-pressure chromatography system (MPLC) on a Redisep RF C18 reverse-phase 43 g column, under stepwise gradient elution with MeOH-H_2_O (from 20:80, 30:70, 40:60, 50:50, 60:40, 80:20 to 100:0; 200 mL for each step). The MPLC 100% MeOH fraction 7 (81.0 mg) was separated using semi-preparative HPLC (Waters Atlantis T3 C18 column: 10 × 250 mm, 5 μm) with a gradient flow solvent system (70–100% MeOH-H_2_O [0.05% trifluoroacetic acid (TFA)] for 20 min, UV 420 nm detection, flow rate: 3 mL/min) to obtain a fraction containing compounds **3**–**5**. The separated fraction (16.0 mg) was further purified using semipreparative HPLC [Waters Atlantis T3 C18 column (10 × 250 mm, 5 μm)], a gradient solvent system (80–100% CH_3_CN-H_2_O (0.05% TFA) to 100% CH_3_CN, 3 mL/min) over 20 min to yield compounds **3** (*t*_R_ = 18.0 min, 4.1 mg), **4** (*t*_R_ = 18.8 min, 3.0 mg), and **5** (*t*_R_ = 16.3 min, 2.2 mg). The structures of the compounds were determined based on nuclear magnetic resonance (NMR) analysis (Supplementary Table S3). Those compounds (**3**–**5**) are identified based on a comparison of previously reported NMR data and molecular formula (Mabit et al., 2017; Heo et al., 2022).

## Results and discussion

### One type I/II hybrid PKS system is responsible for the production of kidamycin and rubiflavinone C-1

Kidamycin (**1**) is a member of the pluramycin family, along with pluramycin A, hedamycin, saptomycin B, and DC92-B, which have anthrapyran aglycones as their core structures. We previously reported the *kid* BGC from *Streptomyces* sp. W2061 (Heo et al., 2022), which is similar in content and organization to the known hedamycin BGC (Bililign et al., 2004). We also confirmed that Kid19, a ketosynthase of type II PKS, is responsible for the biosynthesis of the angucycline core (Heo et al., 2022).

In this study, we inactivated the type I PKS gene (*kid13*), which may be involved in the production of the aglycone side chain moiety. The gene disrupted mutant (Δkid13) results revealed a complete loss of productivity of **1**, photokidamycin (**2**), and rubiflavinone C-1 (**3**), which is a similar production pattern with the type II PKS gene mutant (Δkid19) (Figure 2). This indicated that two types of anthrapyran aglycones with different side chains at C2 can be synthesized from one type I PKS (Kid13) and type II PKS. Collectively, type I PKSs, including Kid13 and Kid14, can supply two types of precursors to type II PKSs (Kid15–19), which are responsible for the synthesis of the two types of anthrapyran backbones. Therefore, we became interested in the mechanism by which the two types of anthrapyran can be synthesized in a hybrid type I/II PKS system.

### Sequence analysis of two type I PKS (Kid13 and Kid14) in the *Kid* BGC

First, *kid* BGC was compared with hedamycin BGC because its structure is similar to that of hedamycin, except for the *bis*-epoxide moiety at the C2 position. The 2-methyl-2,4-hexadiene moiety at the C2 position of **3** may also be formed via the same biosynthetic pathway as the *bis*-epoxide side chain of hedamycin. The *bis*-epoxide side chain originates from a 2,4-hexadienyl primer unit synthesized from type I PKS, encoded by *hedT* and *hedU* in the hedamycin BGC (Bililign et al., 2004; Das and Khosla, 2009). Kid13, which corresponds with HedT, is a loading module including the KSQ domain and has a unique dual AT system with the domain architecture consisting of AT_1-1_-KSQ-AT_1-2_-ACP (Figure 1).

**Figure 3:**
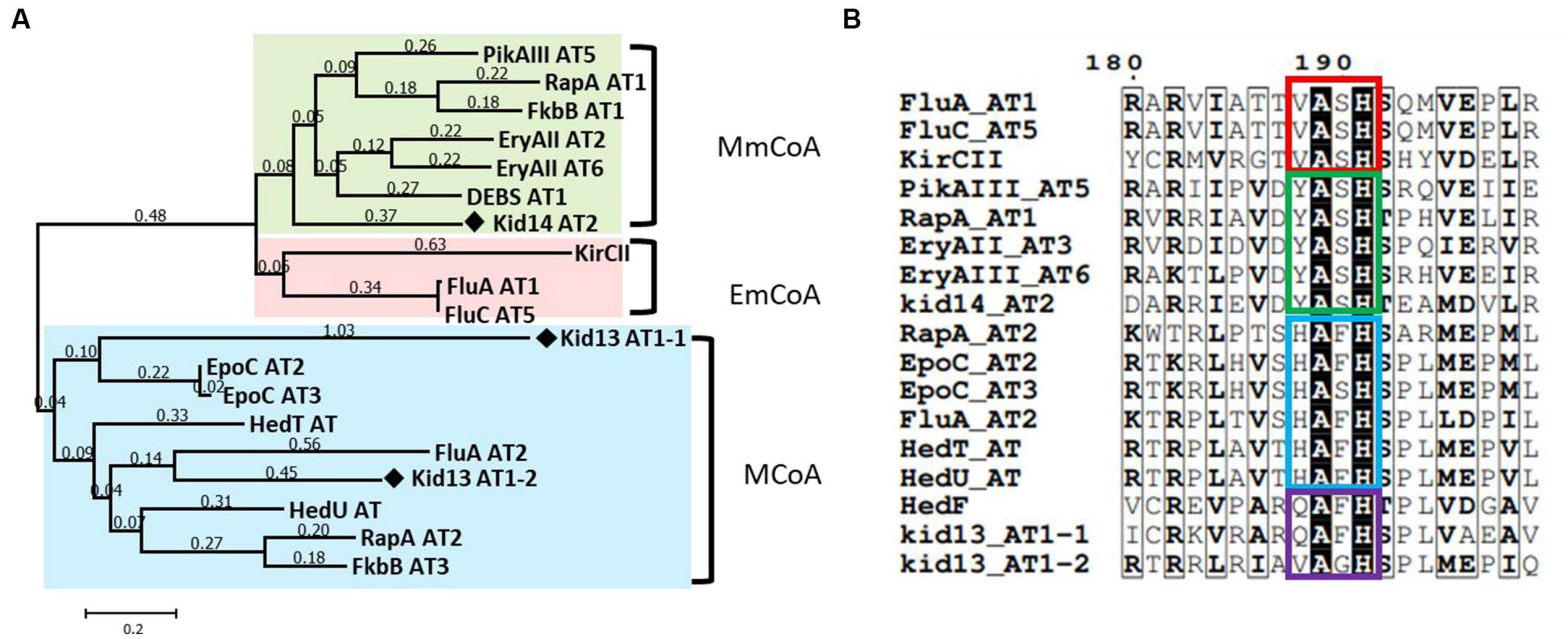
Sequence analysis of three AT domains in Kid13 and Kid14. **(A)** Phylogenetic tree analysis of AT domains in Kid13 and Kid14. **(B)** The conserved motif with color boxes (red for ethylmalonyl-CoA (EmCoA; VASH); green for methylmalonyl CoA (MmCoA; YASH); blue for malonyl CoA (MCoA; HAFH and HASH); and purple for this study).

To determine the substrate specificity of each AT in Kid13, phylogenetic tree analysis was conducted with known type I PKS ATs. Phylogenetic tree analysis based on the amino acid sequences of the AT domains showed that both AT_1-1_ and AT_1-2_ grouped with malonyl-CoA-specific ATs. Interestingly, AT_1-1_ belongs to the malonyl-CoA-specific group but shows a far greater distance in this group (Figure 3A). It is commonly known as the conserved amino acids, such as the “HAFH motif” to predict malonyl-CoA specificity, the “YASH motif” to predict methylmalonyl-CoA, and the “VASH motif” to predict ethylmalonyl-CoA; however, the “HASH motif” is used to predict both malonyl-CoA and methylmalonyl-CoA (Dunn and Khosla, 2013). Specifically, AT_1-1_ has “QAFH” while AT_1-2_ has “VAGH,” and these motifs are not typical sequences found in the ATs of KSQ type loading modules, so no precise prediction of its substrate preference can be made (Figure 3B; Supplementary Figure S2). These data indicated that the substrate specificity of ATs in Kid13 is not predictable, Kid13 may have a different loading mechanism compared with general type I PKS, and the unusual dual AT architecture of Kid13 can be attributed to the synthesis of the two types of products from one gene cluster. Therefore, we hypothesized that each AT has a different substrate specificity and can load two types of precursors to produce two types of anthrapyran, i.e., **1** and **3**.

In addition, the second module encoded by the *kid14* gene was also different from that encoded by hedamycin (*hedU*). Kid14 contains a methylmalonyl-CoA-specific AT and an additional reducing domain, the ER domain (Figure 1). The second module (Kid14) was estimated to synthesize side chains different from HedU, and the second module of hedamycin biosynthesis was proposed to synthesize hexanoate units by iteratively extending two malonyl-CoA units (Das and Khosla, 2009). It has also been estimated that the C15 methyl residue of the hexanoate side chain in hedamycin is introduced by a methyltransferase; however, the methylation reaction has not yet been proven. However, the C15 methyl residues of **1** and **3** may originate from methylmalonyl-CoA because the AT2 domain of Kid14 has a methylmalonyl-CoA conserved motif, whereas the AT domain of HedU has a malonyl-CoA conserved motif (Figure 3). Additionally, we previously reported that all three methyltransferases in *kid* BGC are involved in the biosynthesis of amino sugars, such as N,N-dimethylvancosamine and angolosamine (Heo et al., 2022). Collectively, these results suggest that there is an important difference between **1** and hedamycin in side-chain biosynthesis.

### Dual AT system of Kid13 involved in the formation of two different side chains

**Figure 4:**
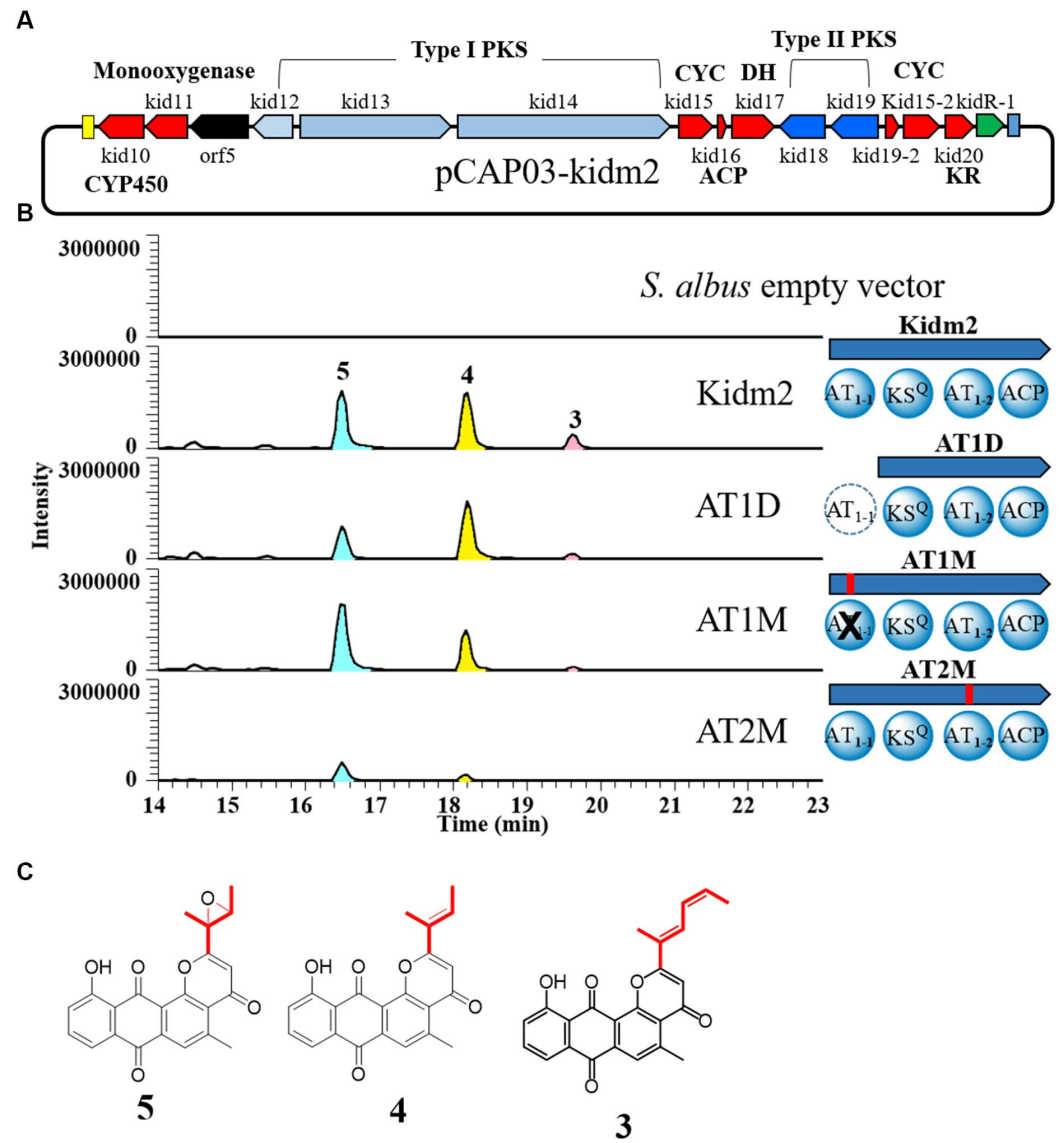
In-frame deletion and site-directed mutagenesis of AT domains of loading module (Kid13). **(A)** The region of minimal PKS genes cloned (pCAP03-Kidm2) and inserted on the chromosome of *S. albus* (Kidm2) for the production of the anthrapyran aglycone. **(B)** Comparative LC-MS extracted ion chromatograms produced from the heterologous expression in recombinant *S. albus* and domain organization of recombinants (AT1D, AT1M, and AT2M) of the Kid13 module. AT1D, the AT_1-1_ domain marked with a dotted circle, was inactivated by an in-frame deletion in the heterologous *S. albus* (Kidm2) chromosome (Supplementary Figure S3). AT1M and AT2M, the AT_1-1_ and AT_1-2_ domains marked with a cross, were inactivated via site-specific mutagenesis, respectively. **(C)** Chemical structures of 4 and 5.

We attempted to identify the role of the dual AT system in heterogeneous strains with a minimal PKS region, containing biosynthetic genes for anthrapyran aglycone. The minimal PKS region was cloned using TAR methods (Yamanaka et al., 2014; Kouprina and Larionov, 2016). The selected heterologous recombinant Kidm2 strain was inserted into hybrid type I/II PKS genes (*kid12* to *kid20*), CYP450 (*kid10*), monooxygenase (*kid11*), and a regulatory gene (*kidR-1*) on the chromosome of *Streptomyces albus* (Figure 4A; Supplementary Figure S1). The Kidm2 strain produced three new peaks compared with those of the parent *S. albus*. New peaks were identified as aglycones [kidamycinone (**4**) and epoxykidamycinone (**5**)] of **1** and **3** (Figure 4; Supplementary Table S3). The ^1^H NMR spectral data for **4** isolates in this study were identical to those described in the literature (Mabit et al., 2017). The other aglycone (**5**) had an epoxide bond at the C14,16 positions. The structure was elucidated by the upfield shift of the oxygenated carbon NMR signal (*δ*_C_ 57.6 and *δ*_C_ 62.0) compared with *δ*_C_ 127.5 and *δ*_C_ 134.7 in **4**, and the degree of unsaturation was 15 (Heo et al., 2022). The presence of **5** may form an epoxide state, in which a reduction in C–C bonds occurs at C14–16. The reduction reaction is estimated to involve Kid10, which showed a high similarity to the HedR involved in introducing the *bis*-epoxide group of hedamycin (Bililign et al., 2004).

This result demonstrated once again that the production of two types of anthrapyran aglycones can be achieved by cloning the hybrid type I/II PKS region. Therefore, we focused on the two AT domains in Kid13 and aimed to determine what happens to the production of the aglycones through inactivation experiments in the two AT domains of Kid13. Each AT in Kid13 was inactivated via site-directed mutagenesis by replacing serine, an active amino acid of AT, with alanine (Hans et al., 2003). The first step in site-directed mutagenesis was performed by inserting a synthetic DNA fragment with a mutation in each AT domain using an *in vitro* CRISPR-Cas9 reaction (She et al., 2018) on the pCAP03-kidm2 vector (Supplementary Figure S3). Mutations in pCAP03-kidm2, pCAP03-AT1M, and pCAP03-AT2M were confirmed by the insertion of restriction enzyme sites SphI and FspI, respectively, at the mutation sites. The final mutant vectors were integrated with the heterologous host *S. albus* to generate the AT1M and AT2M strains (Figure 4B). In addition, because AT_1-1_ is a unique extra domain that cannot be found in a general KSQ-type loading module, an in-frame deleted construct of the AT_1-1_ domain (AT1D) was generated for comparison with the parent strain (Figure 4B). The growth of mutant strains in the M2 medium was normal and comparable to that of the parent Kidm2 strain. Both the in-frame deletion (AT1D) and inactivated mutants (AT1M) of AT_1-1_ showed that short-chain molecules (**4** and **5**) were still produced, and the production of long-chain molecules (**3**) was markedly reduced to only the MS detection level (Figure 4B). The production levels of short-chain molecules (**4** and **5**) in AT1D and AT1M were lower than those of the parent construct. This indicated that malonyl-CoA for short-chain molecules (**4** and **5**) can be primarily loaded by AT_1-2_, and ethylmalonyl-CoA for the long chain of **3** can also be loaded; however, the efficiency is low when AT_1-2_ works alone. Short-chain molecule **4**, compared to **5**, on the AT1M mutant was detected with low production; however, the reason for this cannot be estimated. In contrast, the AT_1-2_ inactivated construct (AT2M) in the heterologous host produced only a small amount of **5**, and the production of **3** was not detected (Figure 4B).

**Figure 5:**
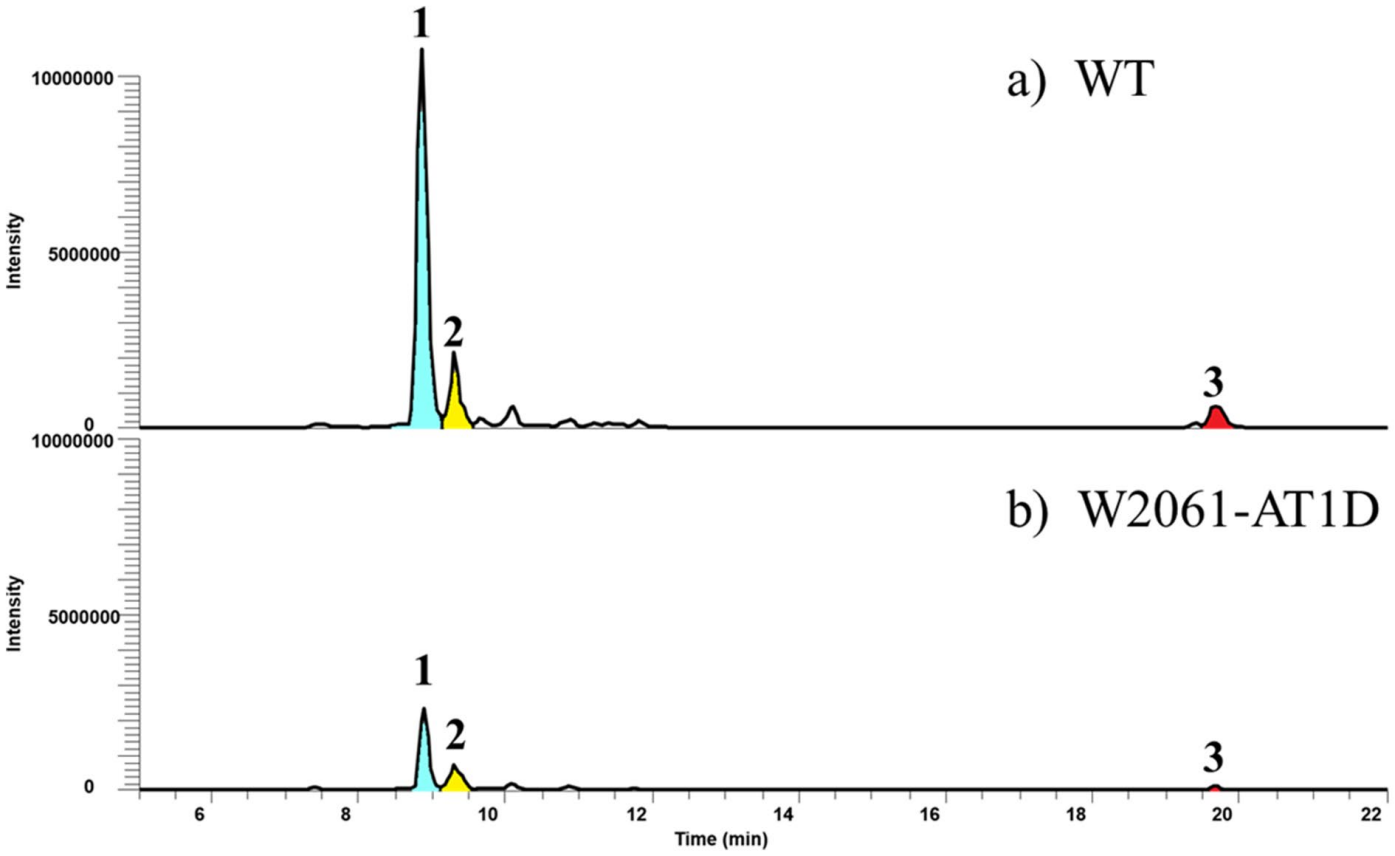
Comparative LC-MS extracted ion chromatograms of culture extracts showing the wild type **(A)** and W2061-AT1D **(B)**, which is an in-frame deleted mutant of AT_1-1_ domain in *Streptomyces* sp. W2061. Production levels are in the same order of magnitude.

In addition, we deleted the AT_1-1_ domain of the Kid13 module in the wild-type *Streptomyces* sp. W2061 strain using the pCRISPomyces-2 system (Cobb et al., 2014; Supplementary Figure S4). The in-frame deletion mutant (W2061-AT1D) was found to have a 942-bp deletion at the 5′ end of the *kid13* gene (Supplementary Figure S4). The W2061-AT1D mutant produced similar metabolic patterns as AT1D, which is a heterologous *S. albus* strain containing the same in-frame deleted AT_1-1_ domain (Figure 5). Glycosylated short-chain molecules (**1** and **2**) are normally produced, although their productivity is marginally lower than that of the wild type. However, it was evidently observed that the production of long chain **3** significantly decreased (Figure 5). These results suggested that AT_1-2_ is primarily involved in the transfer of malonyl-CoA for short-chain molecules and collaborates with AT_1-1_ in the efficient transfer of ethylmalonyl-CoA for long chains.

**Figure 6:**
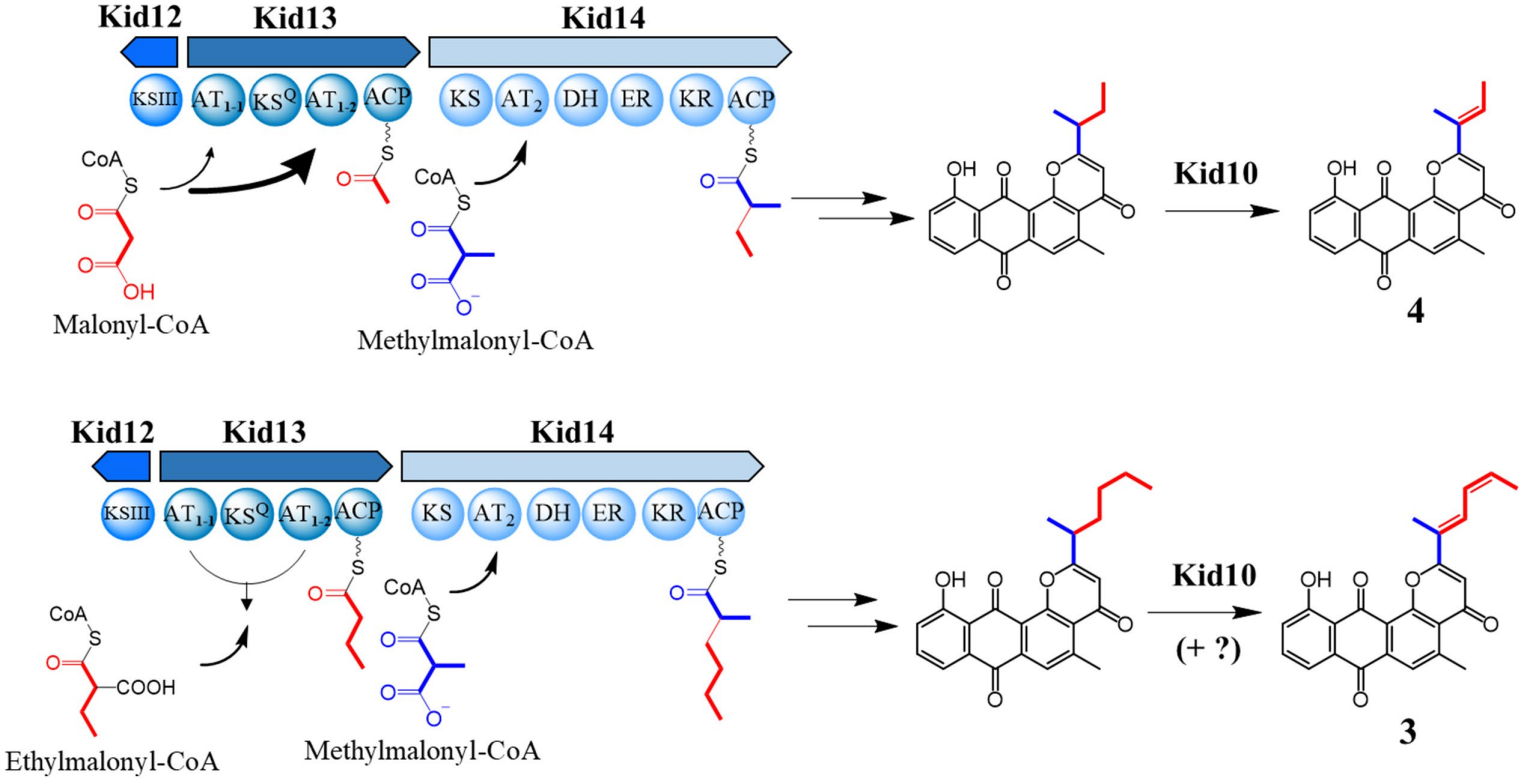
The proposed biosynthetic mechanism for the production of two different side chains of anthrapyran aglycone with an unusual dual AT system in Kid13.

Taken together, AT_1-2_ is the main acyl-CoA transferase in this PKS system for long and short chains, and AT_1-1_ plays an assistant role, especially for long chains, even if AT_1-1_ appears necessary. However, the production of short chains can be produced on AT_1-2_ alone; however, AT_1-1_ is also required for effective production. Overall, AT_1-2_ primarily transfers malonyl-CoA to Kid13, whereas AT_1-1_ also loads malonyl-CoA, but not as much as AT_1-2_. Ethylmalonyl-CoA for long chains was loaded efficiently when AT_1-1_ and AT_1-2_ coexisted, indicating that AT_1-1_ and AT_1-2_ were simultaneously required to transfer ethylmalonyl-CoA (Figure 6). Another possibility is that the low productivity of the two chains in the AT_1-1_ deletion mutants may be the result of protein instability or the discordant architecture of full Kid13 modules caused by the mutation.

The presence of two AT domains in the type I PKS loading module has been reported in biosynthetic pathways such as ajudazol, aurafuron, chondramide, and gulmirecin (Rachid et al., 2006; Frank et al., 2007; Buntin et al., 2010; Schieferdecker et al., 2014). These are loading machinery and have an ACP in the N-terminus, but Kid13 does not have an ACP domain in the N-terminus, indicating a new type of dual AT system. Meanwhile, the AT gene (*hedF*), which is located far from the type I PKS gene (*hedT*) in hedamycin BGC, showed high similarity (79%) and shared the unique conserved motif QAFH with AT_1-1_ (Figure 3B). Notably, HedF is not involved in the chain initiation or elongation of the hexadine side chain (Das et al., 2010). Although it is estimated that the presence of an independent AT protein will lose its function in the chain formation of hedamycin, the AT_1-1_ domain included in type I PKS will maintain its role in the chain formation of **1** and **3**. Therefore, these results are the first to demonstrate that a unique dual AT system of type I PKS can provide two types of precursors to hybrid type II PKS.

In summary, we found that type I PKSs containing an unusual dual AT domain in the loading module (Kid13) synthesized four- or six-carbon intermediates, which are subsequently transferred to type II PKSs in the **4** and **3** biosynthetic pathways, respectively (Figure 6). The presence of unusual loading and extension modules in these type I PKSs (Kid13/Kid14) led to the incorporation of two different primer units, C4 and C6, which were significantly different from the chain initiation of hedamycin and fredericamycin. The dual domains (AT_1-1_ and AT_1-2_) worked together to load two different primer units. The AT_1-2_ domain primarily loaded both malonyl-CoA and ethylmalonyl-CoA for **4** and **3**; however, the AT_1-1_ domain cooperated to load ethylmalonyl-CoA efficiently for **3** by the AT_1-2_ domain.

## Data availability statement

The datasets presented in this study can be found in online repositories. The names of the repository/repositories and accession number(s) can be found in the article/Supplementary material.

## Author contributions

KH: Investigation, Methodology, Visualization, Writing – original draft, Writing – review & editing. BL: Data curation, Investigation, Methodology, Visualization, Writing – original draft, Writing – review & editing. GH: Data curation, Methodology, Writing – review & editing. BP: Investigation, Methodology, Writing – review & editing. J-PJ: Data curation, Formal analysis, Methodology, Writing – review & editing. BH: Conceptualization, Formal analysis, Project administration, Writing – review & editing. J-HJ: Formal analysis, Funding acquisition, Project administration, Resources, Writing – review & editing. Y-SH: Conceptualization, Funding acquisition, Project administration, Writing – original draft, Writing – review & editing.
