# Supplementary material for: A unique dual acyltransferase system shared in the polyketide chain initiation of kidamycinone and rubiflavinone biosynthesis
Source: Front Microbiol. 2023 Oct 27;14:1274358. doi: 10.3389/fmicb.2023.1274358 (PMC10646177; doi:10.3389/fmicb.2023.1274358)
Supplement: Supplementary file 1 [file Data_Sheet_1.DOCX]

**SUPPLEMENTAL MATERIAL**

A unique dual acyltransferase system shared in the polyketide chain initiation of kidamycinone and rubiflavinone biosynthesis

Kyung Taek Heo^1,3^, Byeongsan Lee^1,2,3^, Gwi Ja Hwang^1^, Beomcheol Park^1,2^, Jun-Pil Jang^1^, Bang Yeon Hwang^2^, Jae-Hyuk Jang^1,*^, & Young-Soo Hong^1,*^

^1^ Chemical Biology Research Center, Korea Research Institute of Bioscience and Biotechnology (KRIBB), 30 Yeongudanji-ro, Cheongju-si, 28116, Korea

^2^College of Pharmacy, Chungbuk National University, Cheongju-si, 28160, Korea

^3^These authors contributed equally; Kyung Taek Heo, Byeongsan Lee.

*** Correspondence:**Jae-Hyuk Jang, [jangjh@kribb.re.kr](mailto:jangjh@kribb.re.kr); Young-Soo Hong, hongsoo@kribb.re.kr

**Table S1. Primers and sgRNA oligomers used in this study.**

| **Primer** | **Sequence (5’-3’)** | **RE** |
| --- | --- | --- |
| Kid4-H1-F | gaaTTCTACCTCTGCTTCCCGGCTC |  |
| Kid4-H1-R | ggtACCCGGGACGCACATCCCGGT |  |
| Kid11_F | ATGACTCGCGTGCTCGTCATCGGC |  |
| Kid11_R | TCATCGTGCGATCTCCTTGGCGTC |  |
| Kid13-H1-F | *gaaTTC*GACTCCCGGGCCGACGGGATC | EcoRI |
| Kid13-H1-R | *ggtacc*GACCGGTCCGGGCTCGGGGCCGA | KpnI |
| Kid13-H2-F | *ctgcaG*ACTCCCGGATGGCCGTCACTC | PstI |
| Kid13-H2-R | *aagCTT*CAGCGAGCCGAGCCACAGC | HindIII |
| Kid13sc_F | GCTGGAGGCGGGGGAGTGCTCG |  |
| Kid13sc_R | CGGCTTCGCGGGGTGAGATGCC |  |
| KidR1_scF | GGCAGTCGCAGGCGCTCCAGTC |  |
| KidR1_scR | CAGTTCGCCGCCCTCCAGCACCAG |  |
| AT1D_scF | GCGGGCGCCCAGAGATCGTGTCCT |  |
| AT1D_scR | CGTCGGTCGTCGGGTCGGGTTCTG |  |
| AT1-2_scF | ACCCGCGGCGAAGGTCTCACC |  |
| AT1-2_scR | GGCTCGCGCCGGAAGGGGTAGG |  |
| **sgRNAs** | **Sequence (5’-3’)** |  |
| Kid10_sg1 | GCGAUCUGGUCAACAUGGUC |  |
| Kid10_sg2 | UGGUGACGAUCGGCGACGUG |  |
| Kid17_sg1 | GGAUCCCCGCCGCCUACGGC |  |
| Kid17_sg2 | GACUGCUGCUGGUGGUGGCC |  |
| AT1-1D_sg1 | CGCUCUUGCCCCCGACCACC |  |
| AT1-1D_sg2 | AGGCCGCAGCGGCCGUGGAG |  |
| AT1-1_sg1 | GUGCGCCCGGUGGCCCUGAU |  |
| AT1-1_sg2 | GCACAGCGUCGGGGAACUCG |  |
| AT1-2_sg1 | AUGGCGCCGGACCUGCTGCU |  |
| AT1-2_sg2 | GCGCGGCGGUGAUCUCGCCC |  |
| **Capture arms** | **Sequence (5’-3’)** |  |
| Kidam_f2 | catggtataaatagtggcGGCCATCCCGCTCAGGGCGGCCTCCAGCTCCTCGGGGGTGTCCATGTACC*ttaattaa*AATCCAGCTACGCGGTCAGCGACGTCCTGGTGCTGGAGGGCGGCGAACTGtatgtcgaaagctacata | PacI |

**Table S2**. Bacterial strains and plasmids used in this work

| **Plasmid or strain** | **Description** | **Source or references** |
| --- | --- | --- |
| pKC1139 | Temperature sensitive *E. coli-Streptomyces* shuttle vector, Apr^R^, oriT (RK2), pSG5 rep | Kieser et al. |
| pFD-NEO-S | pUC19 containing neo (Kanamycin resistance gene) | Denis and Brzezinski |
| pKC1139-Kid13H12neo | pKC1139 carrying two franking arms amplified from *Streptomyces* sp. W2061 and neo amplified from pFD-NEO-S to knock out *kid13* gene | This study |
| pCAP03-acc(3)IV | URA3 under the control of PADH. Kan^R^. Used for transformation-associated recombination. | Tang et al. |
| pCAP03-kidm2 | pCAP03-acc(3)IV carrying kidamycin minimal PKS (from kid10 to kidR-1) region. | This study |
| pCAP03-AT1M | AT_1-1_ domain inactivation in pCAP03-kidm2 | This study |
| pCAP03-AT2M | AT_1-2_ domain inactivation in pCAP03-kidm2 | This study |
| Kidm2-AT1D | AT_1-1_ in-frame deletion in pCAP03-kidm2 | This study |
| pCRISPomyces-2 | *Streptomyces* CRISP/cas9 vector, Apr^R^, oriT, reppSG5(ts), oriColE1, sSpcas9, synthetic guide RNA cassette | Cobb. et al. |
| AT1DH_TA | T-Blunt vector with editing template (PstI digested fragment from Kidm2-AT1D) for deletion of AT_1-1_ | This study |
| pCRI-AT1D | pCRISPomyces-2 with sgRNA and 2.5kb editing template for deletion of AT_1-1_ | This study |
| *Streptomyces* sp. W2061 | Kidamycin and rubiflavinone C-1 producer | Lee et al. |
| *Streptomyces albus* J1074 | Heterologous expression host | Zaburannyi et al. |
| Δkid13 | *Streptomyces* sp. W2061 with *kid13* gene inactivation by double-cross over | This study |
| AT1D-W2061 | AT_1-1_ domain in-frame deletion in *Streptomyces* sp. W2061 using pCRI-AT1D | This study |
| *E. coli* DH5α | Cloning host | Invitrogen |
| *E. coli* ET12567 | RP4-Tc : : Mu-Km^R^ : : Tn7, Cm^R^, dam^−^, dcm^−^, hsdM^−^ | Kieser et al. |
| ET12567/pUB307 | ET12567 carrying pUB307 (RP1 derivative, Km^R^) | Flett et al. |
| *E. coli* ET12567/pUZ8002 | ET12567 carrying pUZ8002 (*oriT* mobilizing plasmid, Km^R^) | Kieser et al. |

Apr^R^, apramycin; Km^R^, kanamycin; Cm^R^, chloramphenicol

**Table S3. ^13^C (175 MHz) and ^1^H (700 MHz) NMR spectroscopic data for compound 3-5.**

|  | **3*** |  | **4*** |  | **5*** |  |
| --- | --- | --- | --- | --- | --- | --- |
|  | *δ*_C_ | *δ*_H_ (mult., *J* in Hz) | *δ*_C_ | *δ*_H_ (mult., *J* in Hz) | *δ*_C_ | *δ*_H_ (mult., *J* in Hz) |
| 2 | 164.4 |  | 164.4 |  | 168.2 |  |
| 3 | 109.7 | 6.42 (s) | 109.0 | 6.35 (s) | 109.6 | 6.50 (s) |
| 4 | 179.8 |  | 180.0 |  | 179.1 |  |
| 4a | 126.6 |  | 126.6 |  | 126.6 |  |
| 5 | 150.1 |  | 150.0 |  | 149.9 |  |
| 6 | 125.8 | 8.01 (s) | 125.7 | 8.00 (s) | 125.9 | 8.09 (s) |
| 6a | 136.7 |  | 136.2 |  | 136.1 |  |
| 7 | 182.1 |  | 182.2 |  | 181.8 |  |
| 7a | 132.6 |  | 132.5 |  | 132.3 |  |
| 8 | 119.7 | 7.8 (d, 7.4) | 119.6 | 7.79 (d, 7.4) | 119.4 | 7.84 (d, 7.4) |
| 9 | 136.3 | 7.66 (t, 7.9) | 136.6 | 7.65 (t, 7.9) | 136.5 | 7.71 (t, 8.1) |
| 10 | 125.5 | 7.32 (d, 8.3) | 125.5 | 7.33 (d, 8.3) | 125.4 | 7.39 (d, 8.3) |
| 11 | 162.9 |  | 162.8 |  | 162.6 |  |
| 11a | 117.1 |  | 117.0 |  | 116.8 |  |
| 12 | 187.9 |  | 187.7 |  | 187.2 |  |
| 12a | 119.8 |  | 119.8 |  | 119.8 |  |
| 12b | 156.5 |  | 156.5 |  | 156.4 |  |
| 13 | 24.4 | 3.00 (s) | 24.4 | 2.99 (s) | 24.3 | 3.03 (s) |
| 14 | 125.4 |  | 127.5 |  | 57.6 |  |
| 15 | 12.4 | 2.05 (s) | 12.4 | 2.02 (s) | 13.7 | 1.85 (s) |
| 16 | 131.2 | 8.39 (d, 11.9) | 134.7 | 7.47 (d, 7.1) | 62.0 | 3.51 (q, 5.4) |
| 17 | 125.5 | 6.50 (t, 11.3) | 15.3 | 2.01 (d, 7.1) | 14.1 | 1.56 (d, 5.4) |
| 18 | 135.6 | 6.07 (qd, 7.5, 11.5) |  | . |  |  |
| 19 | 14.3 | 2.17 (d, 7.2) | . | . |  |  |
| 11-OH |  | 12.97 (s) |  | 12.89 (s) |  | 12.87 (s) |

* Compound **3**-**5** was previously reported by Heo *et al.*

**Table S4. Percentage of identity/similarity between the amino acid sequences of Kid13 and Kid14 and HedT and HedU from hedamycin BGC.**

| **Gene (domain)** | | **Similarity(%)** | **Identity(%)** |
| --- | --- | --- | --- |
| **Kid13** | **HedT** | **56** | **45** |
| **KS** | **KS** | **65** | **55** |
| **AT1-1** | **AT** | **44** | **34** |
| **AT1-2** | **AT** | **58** | **46** |
| **Kid14** | **HedU** | **54** | **41** |
| **KS** | **KS1** | **70** | **58** |
| **KS** | **KS2** | **52** | **39** |
| **AT** | **AT** | **55** | **39** |
| **DH** | **DH** | **59** | **46** |
| **KR** | **KR** | **73** | **66** |

**Figure S1. TAR based direct cloning strategy for capture of *kid* BGC gene clusters.**

Cosmid library screened via PCR with appropriate primers (kid4-H1-F/ kid4-H1-R and kidR1sc_F / kidR1sc_R; Table S1). A positive clone (12-E) containing kidamycin minimal PKS region was obtained. The capture vector is constructed in one step by Gibson Assembly using synthetic capture arms dsDNA. The capture vector is linearized by the restriction enzyme PacI. Linearized cosmid and linearized specific capture vector were added to yeast cells. Plasmid (pCAP-kidm2) was isolated from the positive clone by colony PCR using two primer pairs (kid11-F/kid11-R and kidR1sc_F/kidR1sc_R; Table S1).


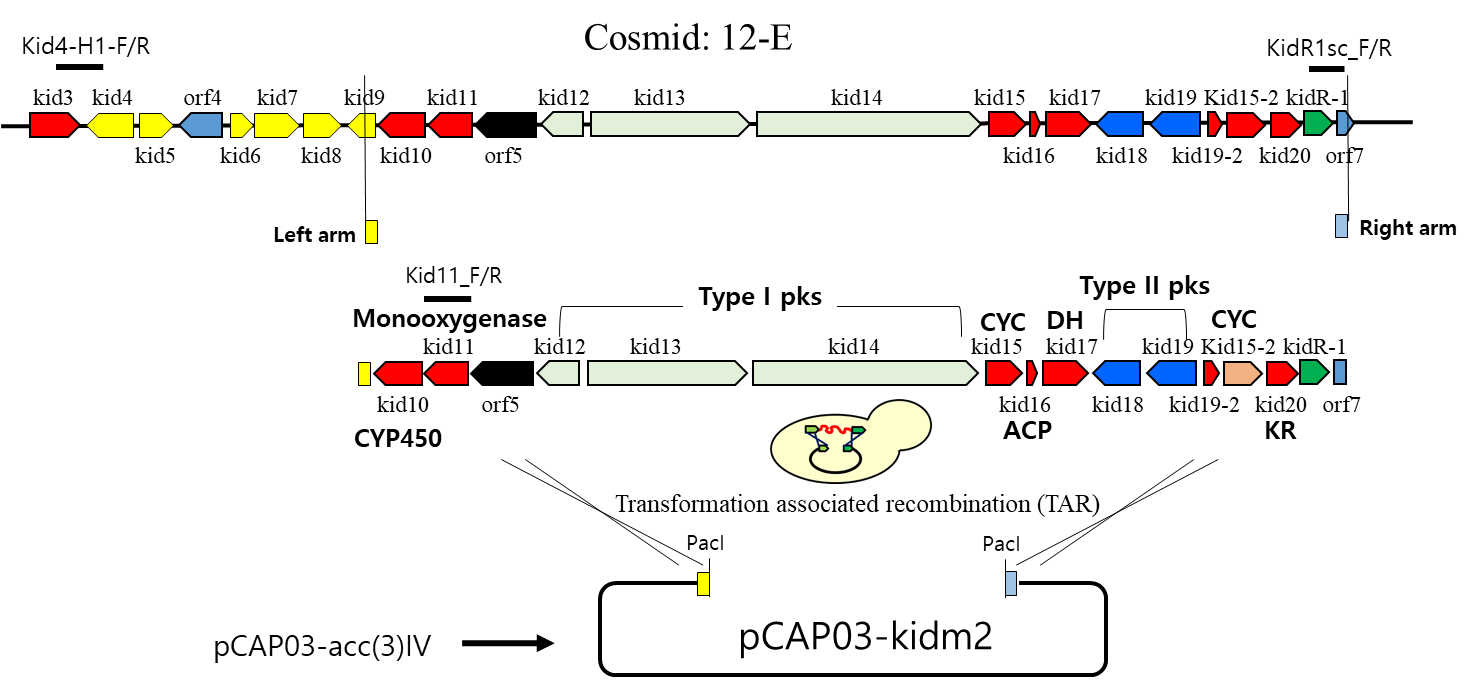


**Figure S2. Amino acid sequence alignment of AT domains from KSQ-type loading modules.**

Colour boxes indicate the substrate-specific conserved motif in AT domains (blue for malonyl CoA(**HAFH**); green for methylmalonyl CoA (**YASH** or **FASH**); red for methoxymalonyl CoA (**TAPH**); purple for this study (**QAFH** and **VAGH**). Amino acid sequences of the KSQ-type ATL were acquired from NCBI GenBank data, and were aligned using the Clustal W method.


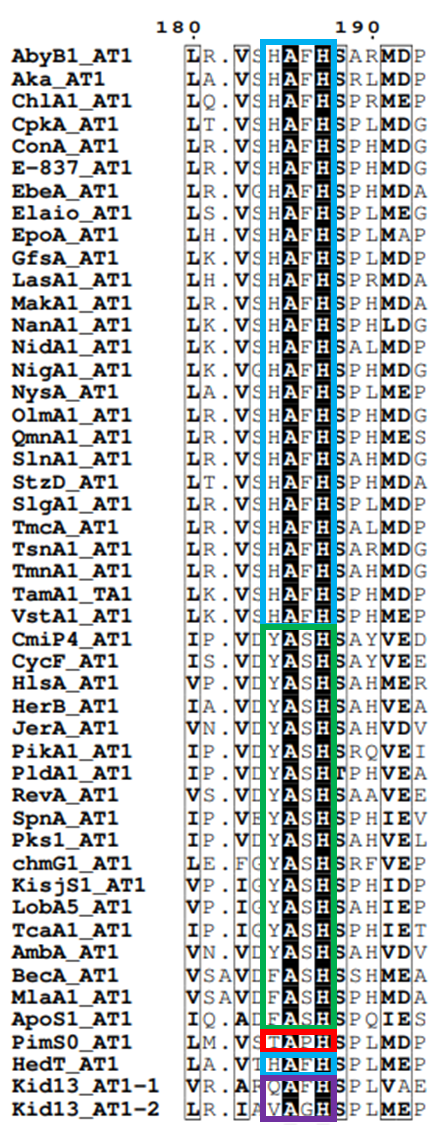
Abyssomicin(JF752342.1), akaeolide(BBOM01000011.1), chalcomycin (AY509120.1), chlorothricin (DQ116941.2), coelimycin (AL645882.2), Concanamycin (DQ149987.1),

E-837 (DQ272520.1), ebelactone (KC894072.1),

elaiophylin (GP697151.1), Epothilone (GU063811.1),

FD-891 (AB469193.1), fostriecin (HQ434551.1),

kijanimicin (EU301739.1), lasalocid (FM173265.1), Lobophorin (KC013978.1), maklamicin (LC021382.1), monensin (AF440781.1), Nanchangmycin (AF521085.1), niddamycin (AF016585.1), nigericin (DQ354110.1),

nystatin (AF263912.1), oligomycin (AB070940.1),

Pimaricin (AJ278573.1), quartromicin (JF970188.1), salinomycin (JN033543.1), streptazone (LC061217.1), streptolydigin (FN433113.1), Tautomycetin (EU035755.1), tetrocarcin (EU443633.1), tetronasin (FJ462704.1), tetronomycin (AB193609.1), Tirandamycin (GU385216.1), versipelostatin (LC006086.1), BE-14106 (FJ872523.1), cremimycin (AB818354.1), cyclizidine (KT327068.1), halstoctacosanolide (AB241068.1),

herboxidiene (JN671974.1), Jerangolid (DQ897668.1),

ML-449 (FJ872525.1), Pikromycin (AF079762.1), pladienolide (AB435553.1), reveromycin (AB568601.1), Apoptolidin (JF819834.1)

**Figure S3. Schematic of CRISPR-Cas9 assisted site-directed mutagenesis and in-frame deletion of the AT domain in pCAP03-kidm2.**

(A) AT_1-1_ in-frame deletion; (B) AT_1-1_ and AT_1-2_ active residue site-directed mutagenesis, and (C) synthetic DNA fragment for inactivation of AT_1-1_ and AT_1-2_

**
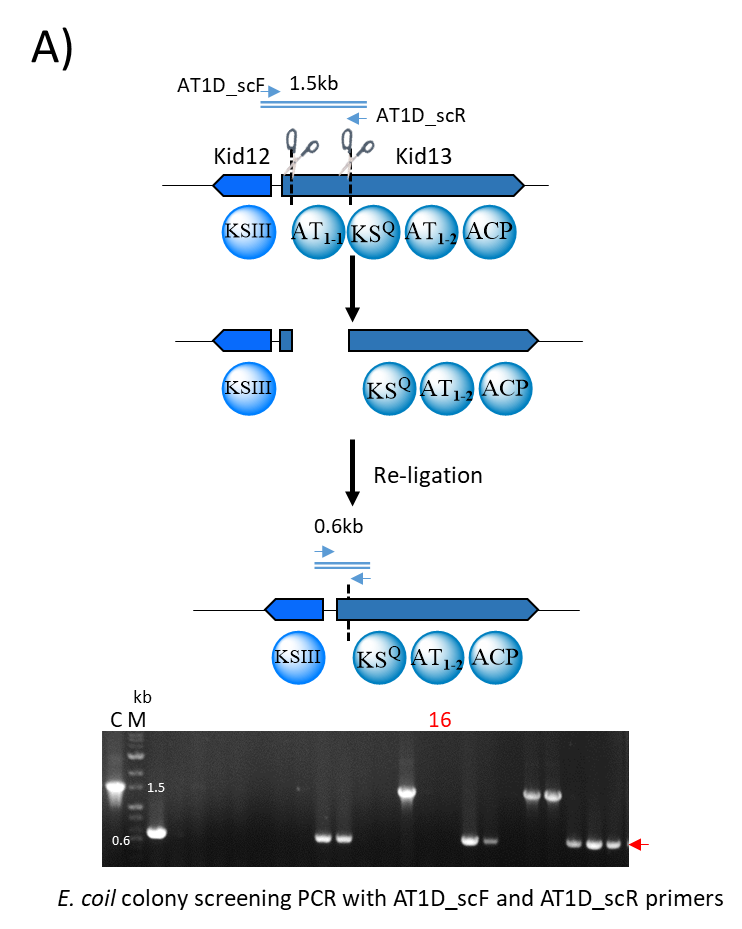
**

**
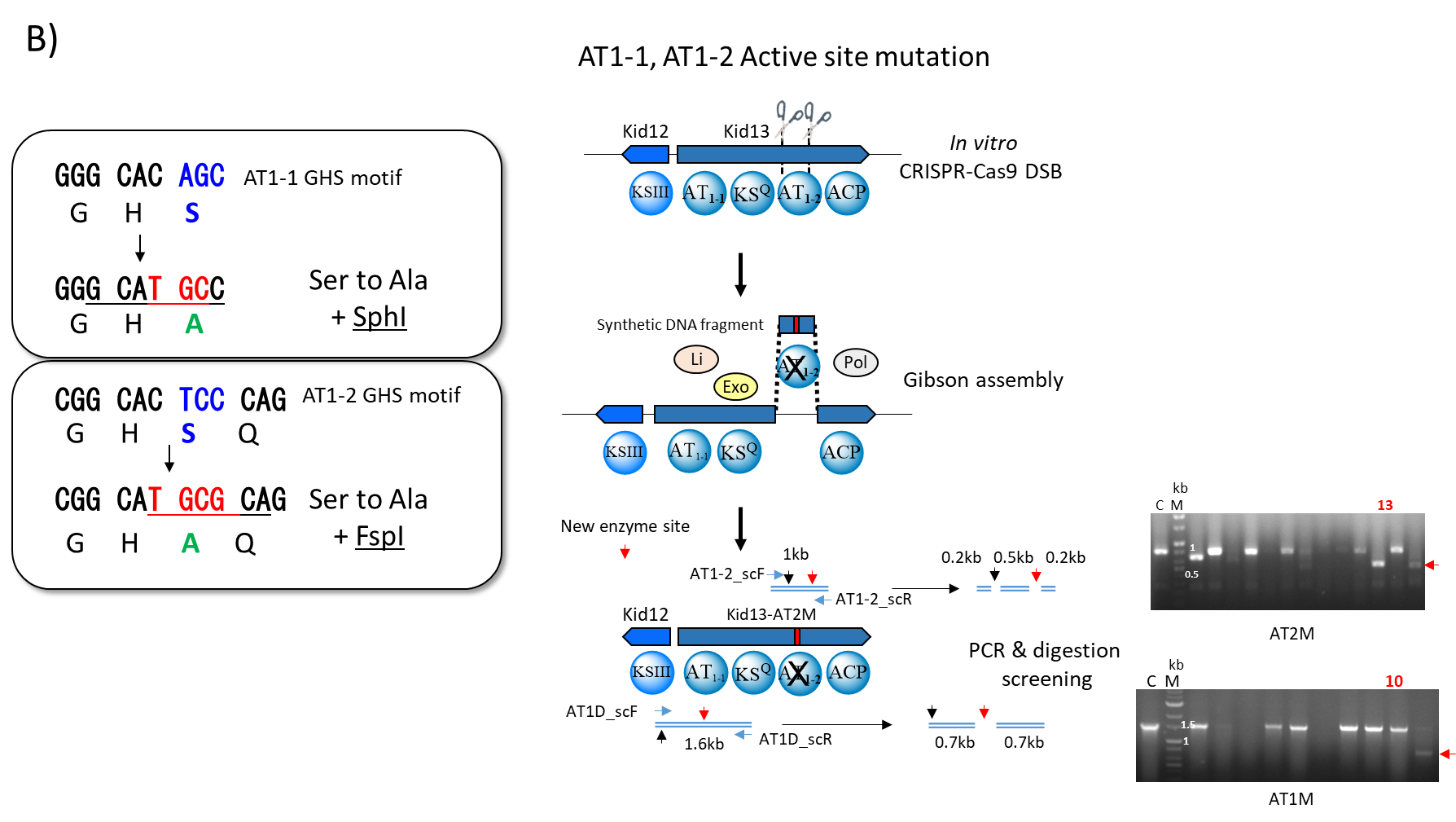
**

**
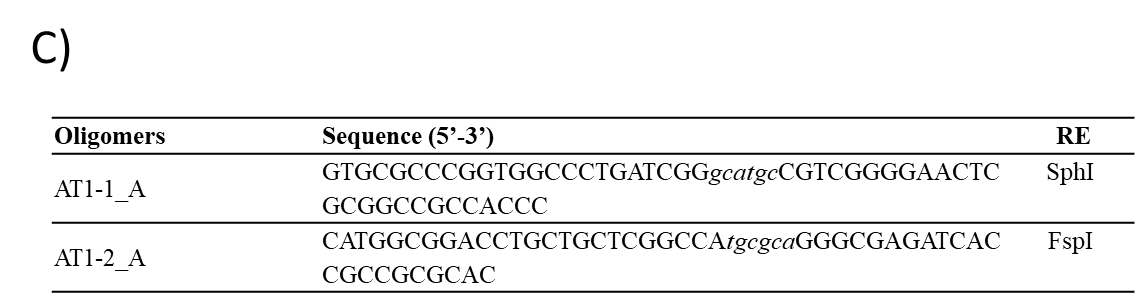
**

**Figure S4. Scheme of CRISPR-Cas9 assisted in-frame deletion of the AT domain in *Streptomyces* sp. W2061 using the pCRISPomyces-2 system.**

**
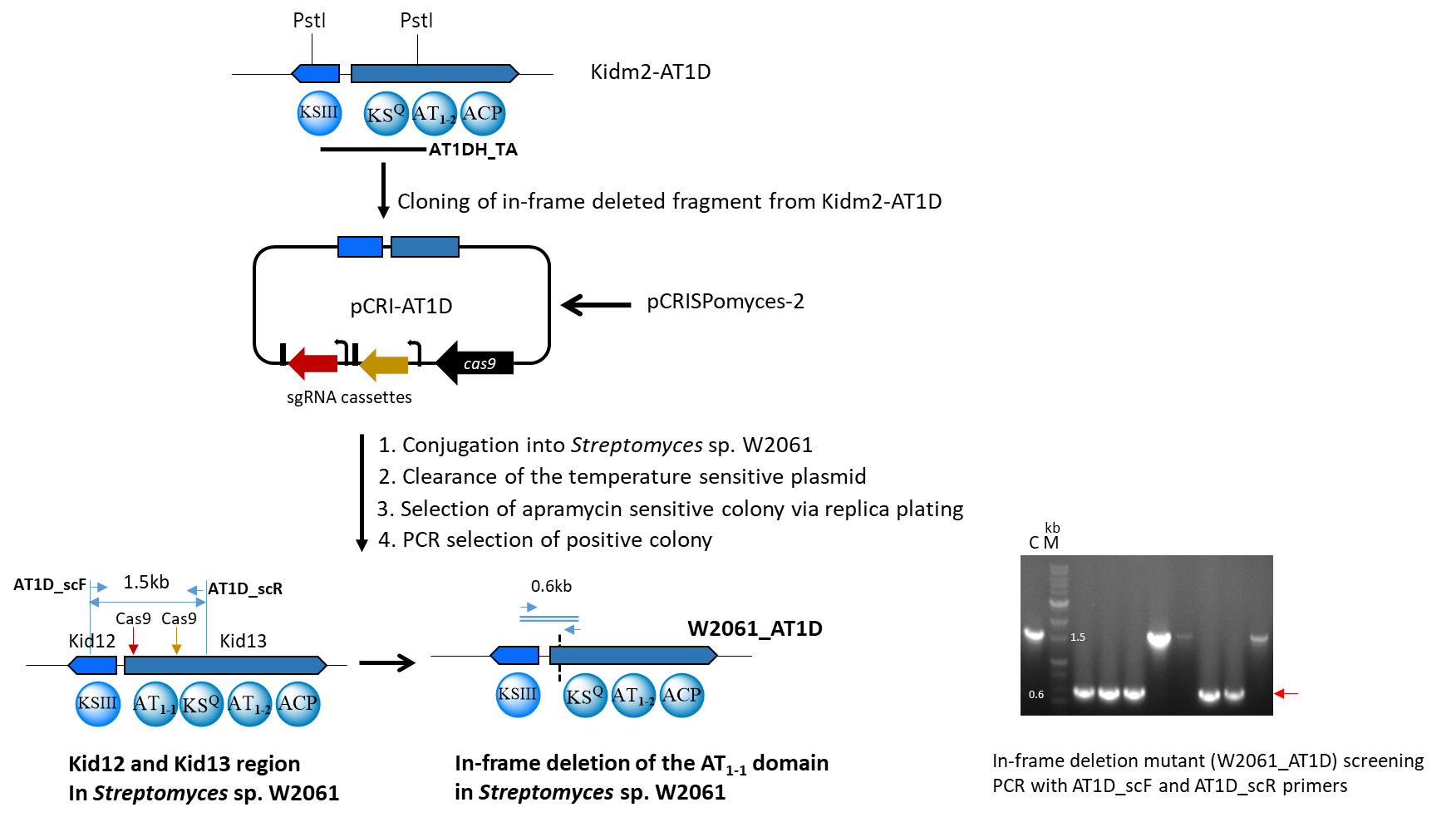
**

**Figure S5.** ^1^H NMR spectrum (700 MHz) of **3** in CDCl_3_-*d*


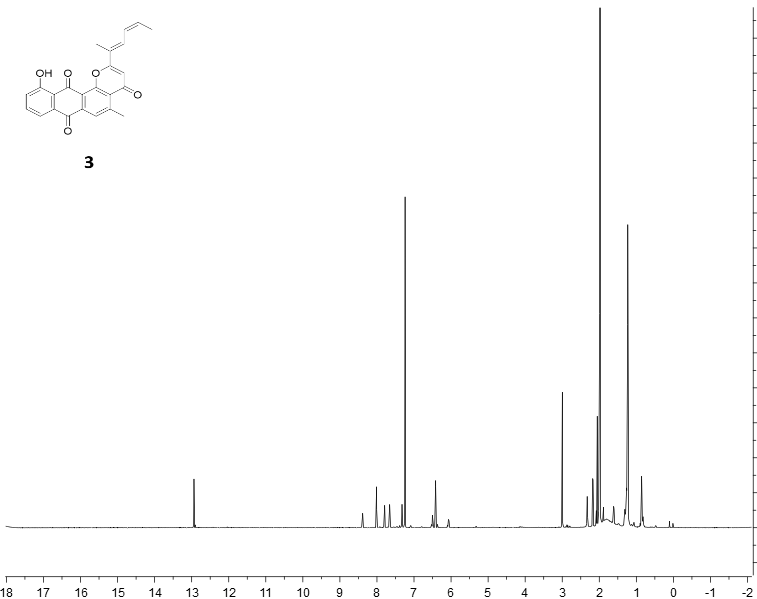


**Figure S6**. ^13^C NMR spectrum (175 MHz) of **3** in CDCl_3_-*d*


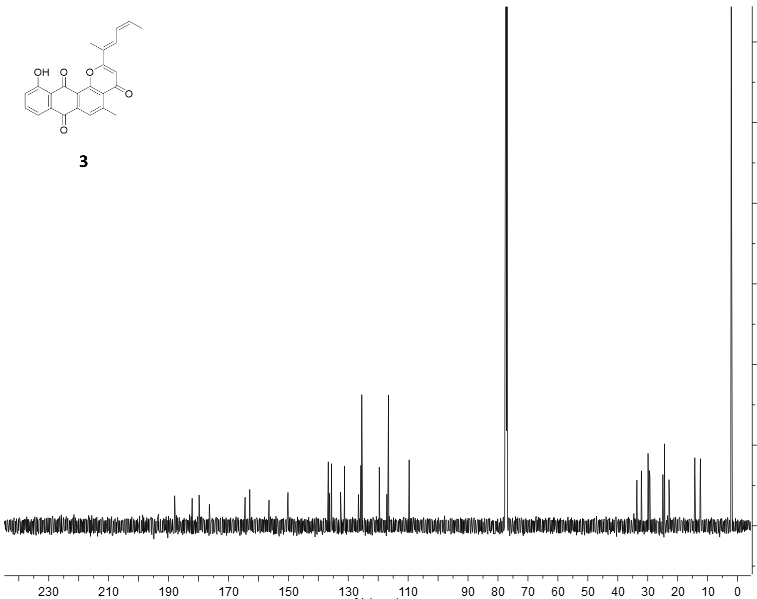


**Figure S7**. DEPT135 spectrum of **3** in CDCl_3_-*d*


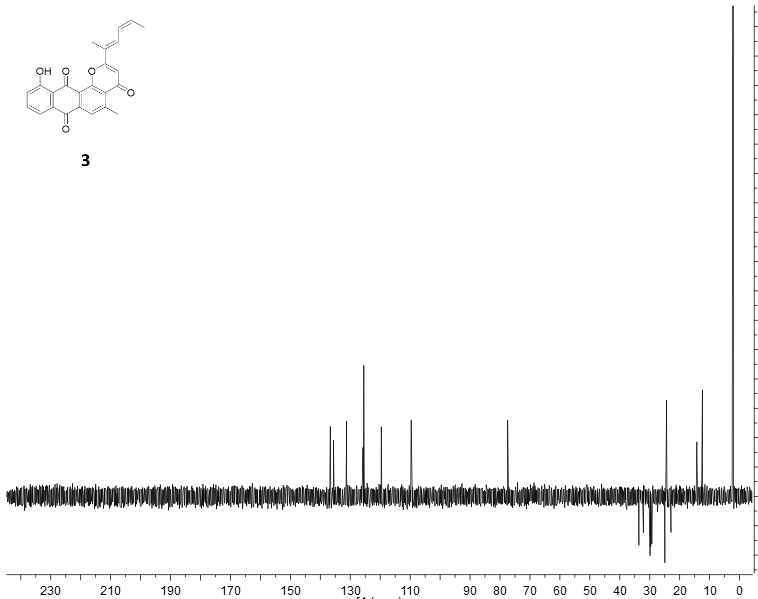


**Figure S8**. COSY spectrum of **3** in CDCl_3_-*d*


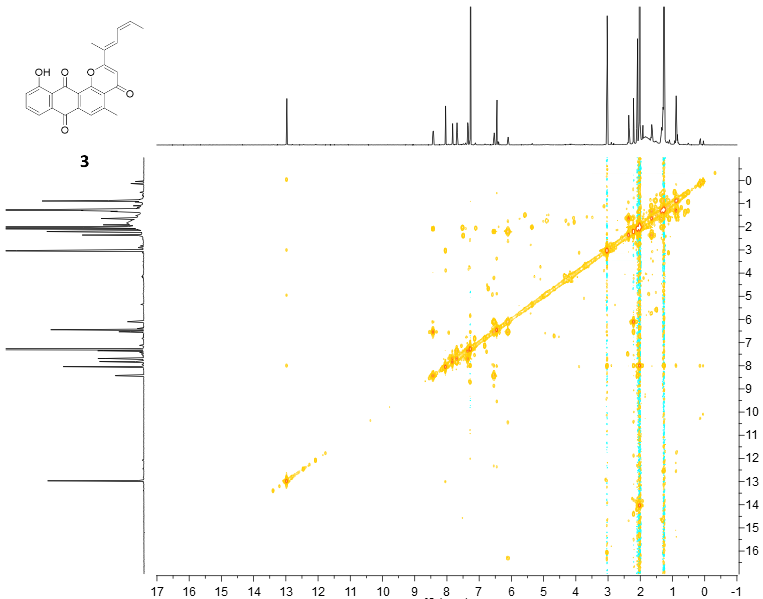


**Figure S9**. HSQC-DEPT spectrum of **3** in CDCl_3_-*d*


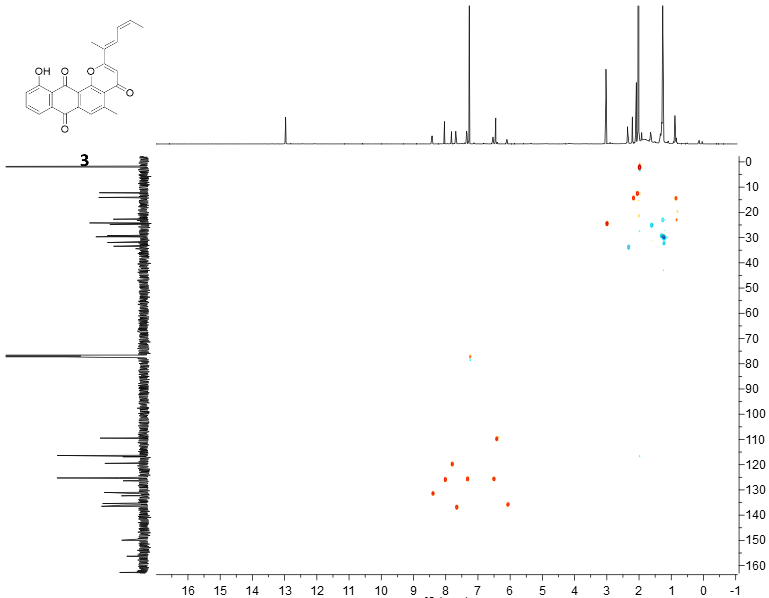


**Figure S10**. HMBC spectrum of **3** in DMSO-*d* CDCl_3_-*d* _6_


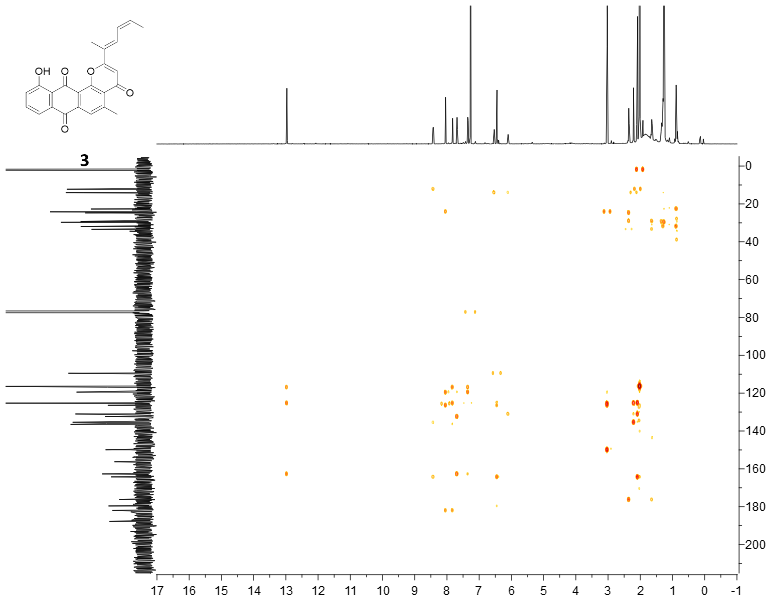


**Figure S11.** ^1^H NMR spectrum (700 MHz) of **4** in CDCl_3_-*d*


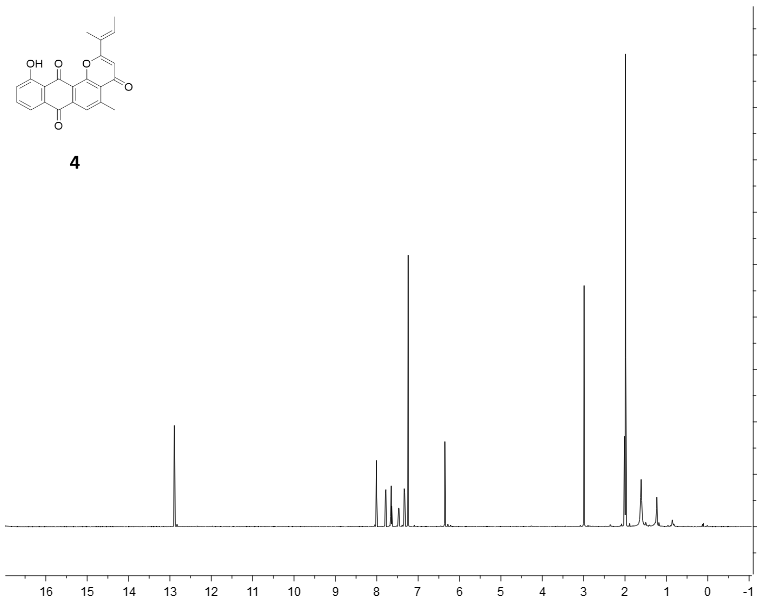


**Figure S12**. ^13^C NMR spectrum (175 MHz) of **4** in CDCl_3_-*d*


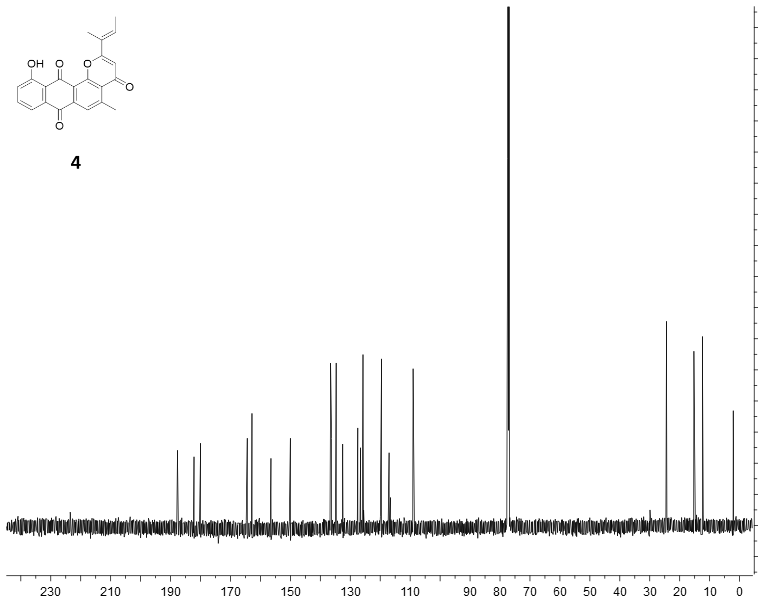


**Figure S13**. DEPT135 spectrum of **4** in CDCl_3_-*d*


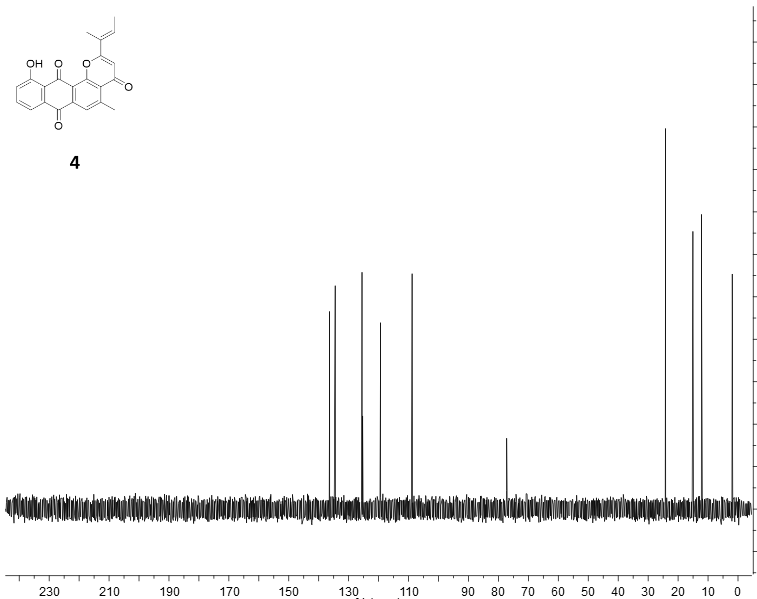


**Figure S14**. COSY spectrum of **4** in CDCl_3_-*d*


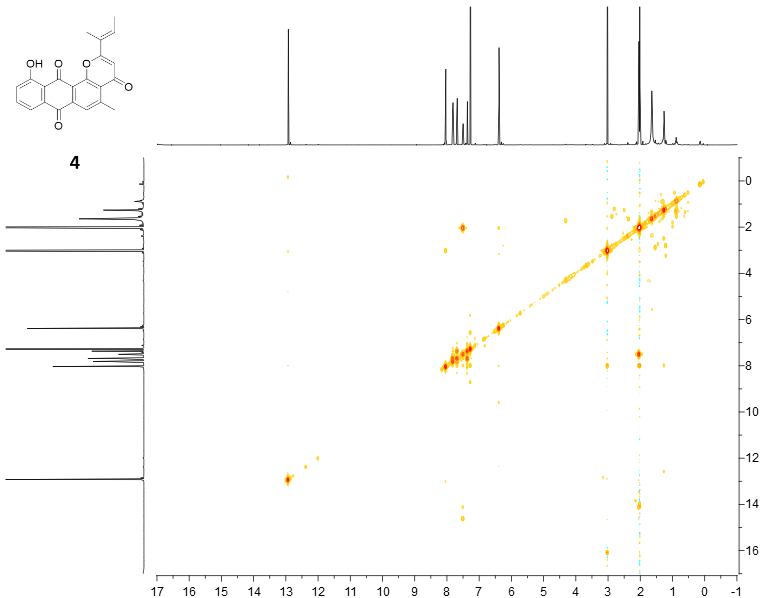


**Figure S15**. HSQC-DEPT spectrum of **4** in CDCl_3_-*d*


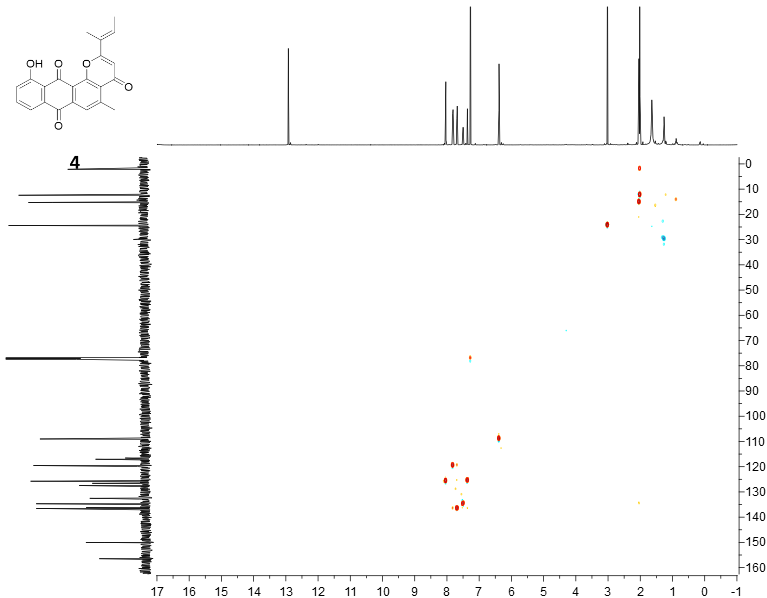


**Figure S16**. HMBC spectrum of **4** in CDCl_3_-*d*


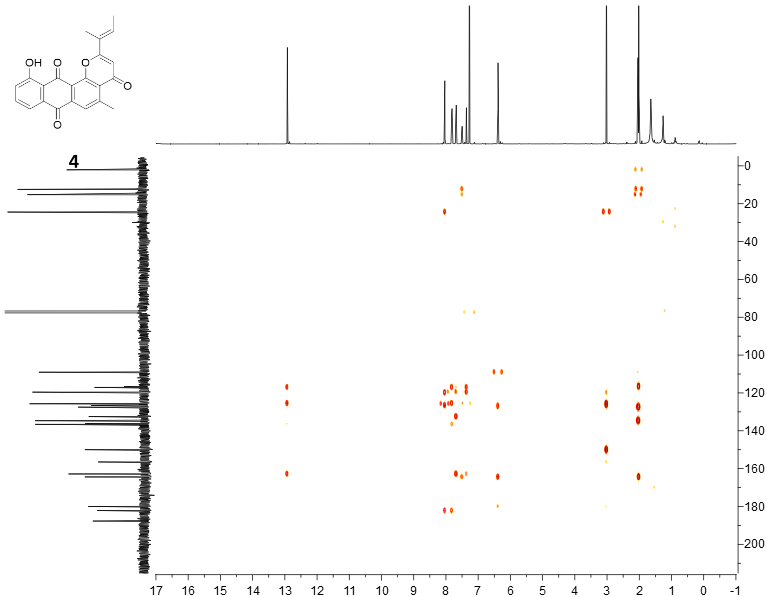


**Figure S17.** ^1^H NMR spectrum (700 MHz) of **5** in CDCl_3_-*d*


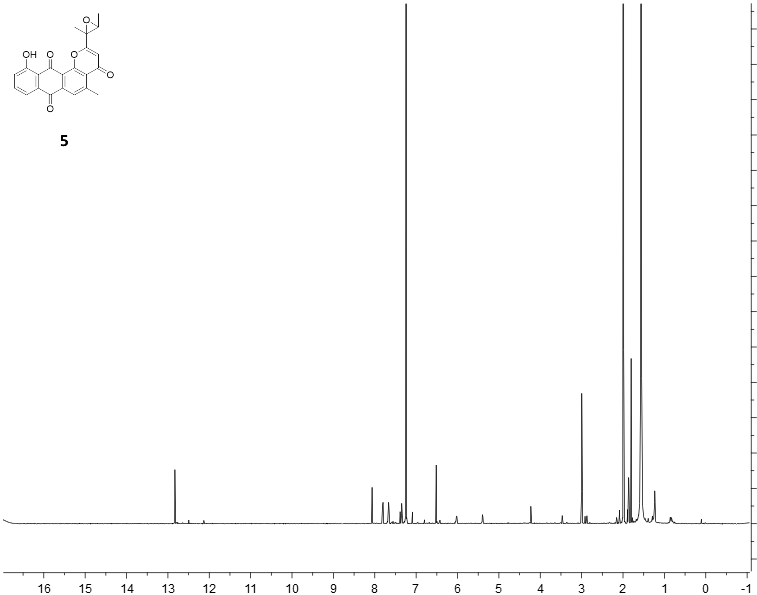


**Figure S18**. ^13^C NMR spectrum (175 MHz) of **5** in CDCl_3_-*d*

5
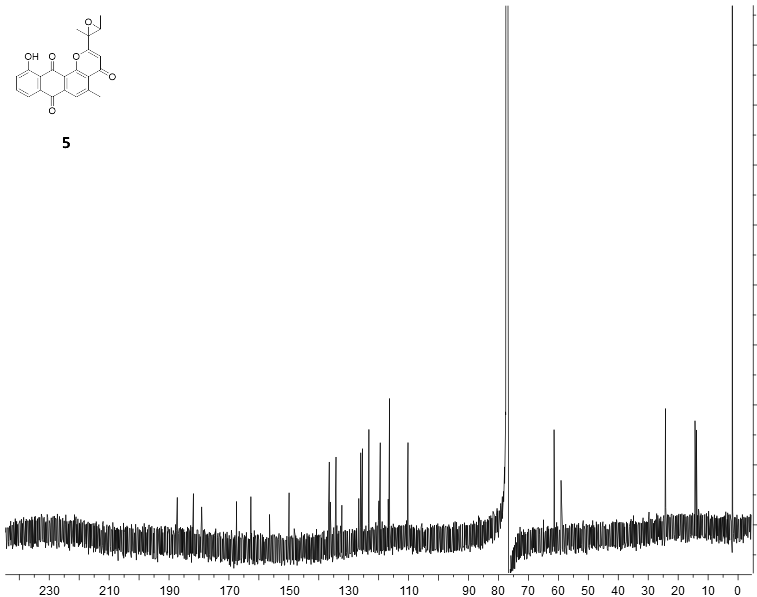


**Figure S19**. DEPT135 spectrum of **5** in CDCl_3_-*d*


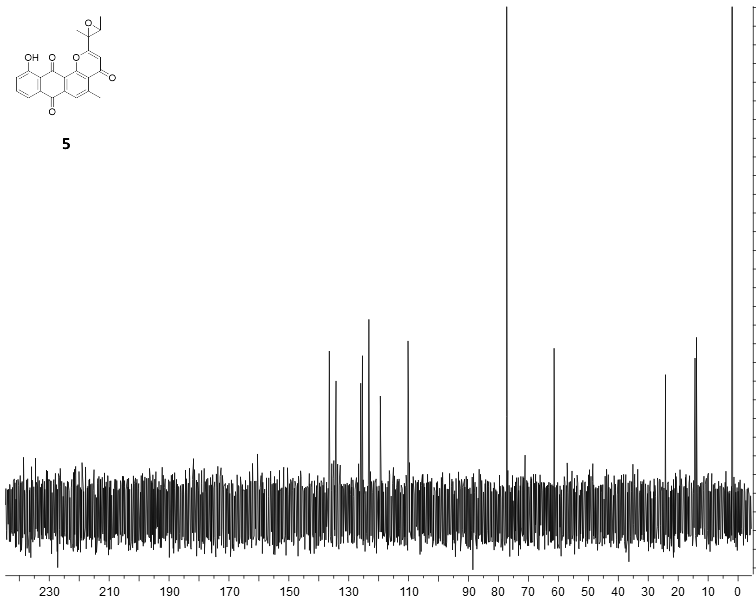


**Figure S20**. COSY spectrum of **5** in CDCl_3_-*d*


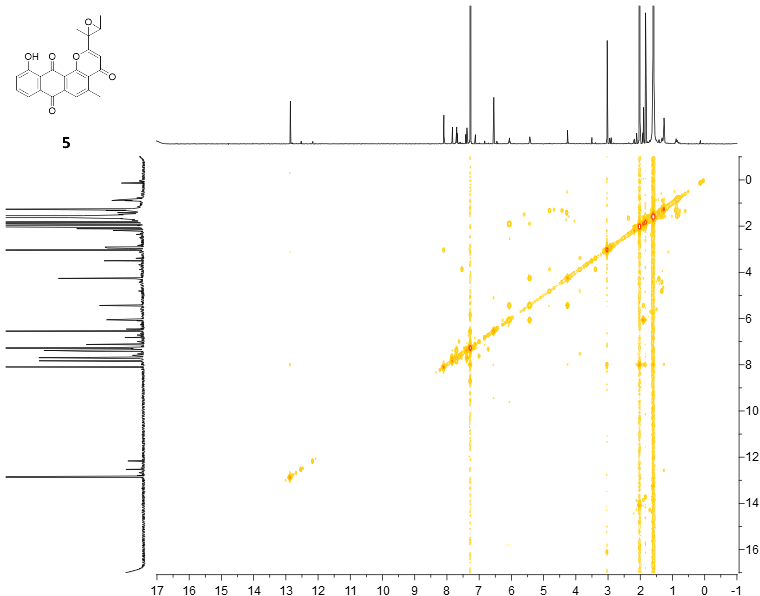


**Figure S21**. HSQC-DEPT spectrum of **5** in CDCl_3_-*d*


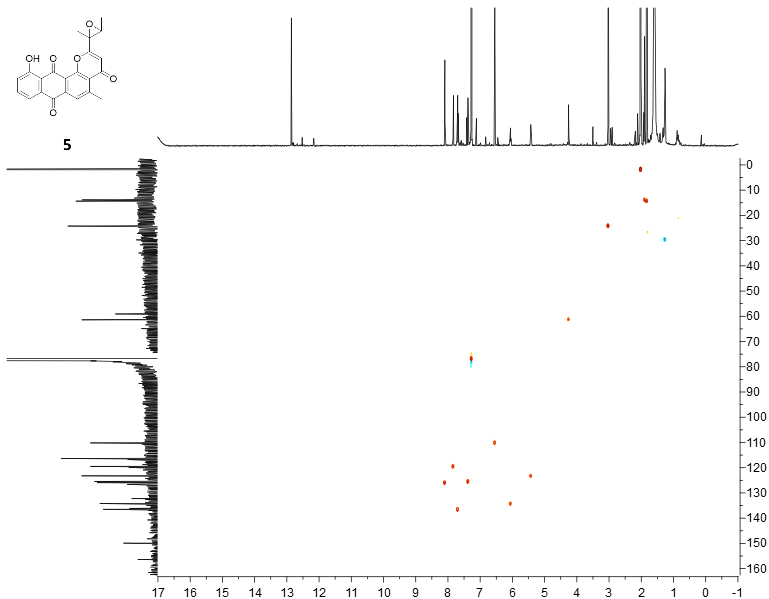


**Figure S22**. HMBC spectrum of **5** in CDCl_3_-*d*


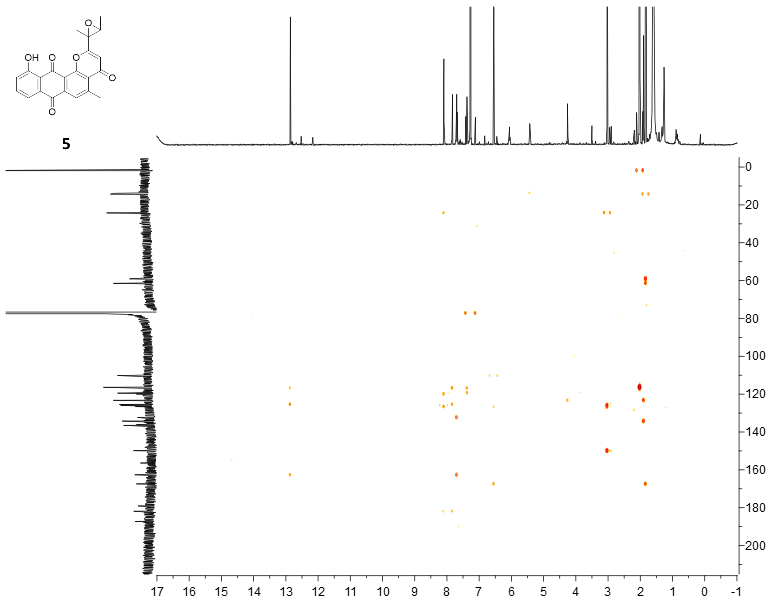


**References**

Cobb, R.E., Wang, Y., and Zhao, H. (2014). High-Efficiency Multiplex Genome Editing of *Streptomyces* Species Using an Engineered CRISPR/Cas System. ACS Synthetic Biology 4, 723-728.

Denis, F., and Brzezinski, R. (1991). An improved aminoglycoside resistance gene cassette for use in gram-negative bacteria and *Streptomyces*. FEMS Microbiol Lett 65, 261-264.

Flett, F., Mersinias, V., and Smith, C.P. (1997). High efficiency intergeneric conjugal transfer of plasmid DNA from *Escherichia coli* to methyl DNA-restricting streptomycetes. FEMS Microbiol Lett 155, 223-229.

Heo, K.T., Lee, B., Jang, J.H., and Hong, Y.S. (2022). Elucidation of the di-c-glycosylation steps during biosynthesis of the antitumor antibiotic, kidamycin. Front. Bioeng. Biotechnol. 10, 985696.

Kieser T., B.M.J., Buttner M. J., Chater K. F., Hopwood D. A. (2000). Practical *Streptomyces* Genetics. John Innes Foundation.

Lee, B., Lee, G.E., Hwang, G.J., Heo, K.T., Lee, J.K., Jang, J.P., Hwang, B.Y., Jang, J.H., Cho, Y.Y., and Hong, Y.S. (2023). Rubiflavin G, photorubiflavin G, and photorubiflavin E: Novel pluramycin derivatives from *Streptomyces* sp. W2061 and their anticancer activity against breast cancer cells. J Antibiot. https://doi.org/10.1038/s41429-023-00643-w.

Tang, X., Li, J., Millan-Aguinaga, N., Zhang, J.J., O'neill, E.C., Ugalde, J.A., Jensen, P.R., Mantovani, S.M., and Moore, B.S. (2015). Identification of Thiotetronic Acid Antibiotic Biosynthetic Pathways by Target-directed Genome Mining. ACS Chem Biol 10, 2841-2849.

Zaburannyi, N., Rabyk, M., Ostash, B., Fedorenko, V., and Luzhetskyy, A. (2014). Insights into naturally minimised *Streptomyces albus* J1074 genome. BMC Genomics 15, 97.
